# The optimal timing for non-culprit percutaneous coronary intervention in patients with multivessel coronary artery disease: A pairwise and network meta-analysis of randomized trials

**DOI:** 10.3389/fcvm.2022.1000664

**Published:** 2022-09-26

**Authors:** Yujia Feng, Shu Li, Sihan Hu, Jing Wan, Hua Shao

**Affiliations:** ^1^Department of Cardiology, Zhongnan Hospital of Wuhan University, Wuhan, China; ^2^Department of Clinical Laboratory, Remin Hospital of Wuhan University, Wuhan, China

**Keywords:** complete revascularization, coronary artery disease, ST-segment elevation myocardial infarction, multivessel disease, culprit-only revascularization, meta-analysis

## Abstract

**Background and aims:**

Recently, several randomized trials have shown that patients with multivessel disease (MVD) often pursue complete revascularization during percutaneous coronary intervention (PCI) to improve their prognosis. However, the optimal time for the non-culprit artery has been controversial. This study aimed to determine the optimal strategy for revascularization in ST-segment elevation myocardial infarction (STEMI) patients with multivessel coronary artery disease (CAD).

**Methods:**

Randomized controlled trials (RCTs) comparing three revascularization strategies [i.e., complete revascularization at the index procedure (CR), complete revascularization as a staged procedure (SR), or culprit-only revascularization (COR)] in STEMI patients with multivessel coronary artery disease were included. We performed both pairwise and network meta-analyses. Network meta-analysis was performed using mixed treatment comparison models.

**Results:**

17 trials with 8568 patients were included. In the network meta-analysis, the most interesting finding was that staged revascularization increased the risk of major adverse cardiac events (MACE) compared with complete revascularization at the index procedure [odds ratio (OR): 1.93; 95% confidence interval (CI): 1.07–3.49]. In the pairwise meta-analysis, complete revascularization reduced the incidence of MACE [risk ratio (RR): 0.62, 95% CI: 0.48–0.79, *p* < 0.001], mainly because it reduced the probability of unplanned repeat revascularization (RR: 0.49, 95% CI: 0.33–0.75, *p* = 0.001). There were no significant differences in all-cause mortality, cardiac mortality, or nonfatal re-myocardial infarction (MI).

**Conclusion:**

Our analysis suggests that complete revascularization should be performed in STEMI patients with multivessel coronary artery disease, and complete revascularization at the index procedure is superior to staged revascularization in reducing the risk of MACE events.

## Introduction

Approximately 50% of patients with ST-segment elevation myocardial infarction (STEMI) have multivessel coronary artery disease (CAD), which is associated with higher mortality and worse prognosis (1). In recent decades, percutaneous coronary intervention (PCI) has become the primary treatment for STEMI. There are three PCI strategies for STEMI patients with multivessel coronary artery disease: (1) complete revascularization (CR), which refers to multivessel PCI at the index procedure; (2) staged revascularization (SR), which refers to culprit artery-only primary PCI followed by staged PCI of non-culprit arteries, either during hospitalization or shortly after discharge; and (3) culprit-only revascularization (COR), which refers to culprit artery-only PCI (2). The optimal timing for revascularization of the non-culprit artery in patients with multivessel coronary artery disease has always been controversial. Clinically, early complete revascularization reduces the risk of cardiac ischemia and improves the long-term outcomes. However, this strategy has certain shortcomings, such as renal insufficiency due to the use of a higher dose of contrast agent, and the additional risk of bleeding when performed as a staged procedure. Previous clinical guidelines recommend against PCI for non-culprit vessels in hemodynamically stable STEMI patients at the time of primary PCI. However, 2021 ACC/AHA/SCAI guideline has changed the recommendation of multi-vessel PCI at the time of primary PCI from Class III (Harm) to Class IIb. Recently, the COMPLETE trial suggested that in STEMI patients with multivessel disease, regardless of the timing of non-culprit artery intervention, complete revascularization is superior to culprit-only revascularization (3). Following the COMPLETE trial, two small RCTs compared the effects of CR and SR in reducing the risk of MACE events and all-cause mortality (4, 5). One study suggested that SR was superior to CR in reducing the risk of all-cause mortality, but there was no significant difference in reducing the risk of MACE events (4). Therefore, we first attempted to compare the effects of revascularization of culprit lesions on the prognosis of patients with multivessel coronary artery disease using pairwise meta-analysis. Furthermore, we will use network meta-analysis to compare the above three revascularization strategies in order to provide some reference for clinical treatment decision making.

## Materials and methods

We followed the 2015 PRISMA extension statement for reporting of systematic reviews incorporating network meta-analyses of health care interventions (6). Since this is a network meta-analysis, neither permission from Institutional Review Board (IRB) nor informed consent from the patient was required. This network meta-analysis was registered in PROSPERO (Complete vs. culprit-only revascularization and the optimal timing for non-culprit PCI in Patients with ST elevation myocardial infarction and Multivessel disease: A Network Meta-Analysis; CRD42020201742).

### Search strategy

We conducted a comprehensive electronic search of the PubMed-Medline, EMBASE, the Cochrane Central Registry of Controlled Trials, Web of Science (SCI-EXPANDED, CPCI-S, and ESCI), Ovid MEDLINE(R) ALL, ClinicalTrial.gov, and major conference proceedings, up to December 2021 with no language restriction using the Medical Subject Heading (MeSH), and the following keywords were used: “myocardial infarction”, “ST elevation myocardial infarction”, “multivessel”, “infarct related”, “non-infarct related”, “culprit”, “non-culprit”, and “randomized”. References of the retrieved articles and previous meta-analyses were also reviewed. We used the wild-card term “^*^” to make the search more sensitive. All the search strategies are provided in the appendix (Supplementary Table 1). Two reviewers (Yujia Feng and Shu Li) independently evaluated each article. A time filter was used to filter articles from 1999 to 2021. The full text of the remaining articles were obtained, and each article was evaluated separately by the same two researchers as before, as well as quality assessment and data extraction. We will discuss with a professor (Jing Wan) to resolve disagreements.

### Eligibility criteria

Studies selected must meet the following criteria: (1) Included STEMI patients with multivessel coronary artery disease, stable hemodynamics, and low anatomical complexity; (2) RCTs that compared any combination of CR, SR, and COR revascularization strategies; (i.e., the trials could compare two or more strategies) (3) Follow-up results data have been reported. None of the trials included in this meta-analysis evaluated patients with hemodynamic instability due to heart failure or shock.

In the pairwise meta-analysis, we compared complete revascularization (i.e., complete revascularization at the index procedure or staged revascularization) to culprit-only revascularization. In the network meta-analysis, the revascularization strategies in these trials were divided into three groups: complete revascularization at the index procedure, staged revascularization, or culprit-only revascularization. We will use network meta-analysis to compare the impact of these three revascularization strategies on patient outcomes.

### Data extraction

Studies that met the criteria were reviewed and the following data were extracted: (1) name of RCTs; (2) year of publication; (3) study design; (4) sample size; (5) patient inclusion and exclusion criteria; (6) interventional strategies and the number of participants with each intervention strategy; (7) mean follow-up months; (8) clinical outcomes (i.e., its definition and results); (9) conclusion; (10) NCT number, if available. The number of events that occurred in each group in each trial was tabulated.

### Outcomes and definitions

The primary outcome of this analysis was major adverse cardiac events (MACE) considered as per-study definition (Supplementary Table 2). Other outcomes included all-cause mortality, cardiac mortality, nonfatal re-MI, unplanned repeat revascularization, re-hospitalization, heart failure, stroke, major bleeding, contrast-induced nephropathy, and stent thrombosis. When a study had both short- and long-term follow-up data, we focused only on the latter. The definitions of COR, CR, and SR have been described previously in the introduction.

### Quality assessment

The included studies were assessed for the risk of bias by the same two researchers using the Cochrane Quality Assessment Tool for RCTs 1.0 (ROB 1.0). ROB 1.0 contains 7 criteria for quality assessment, the risk of bias for each study will be assessed as “low”, “high”, or “uncertain.”

### Statistical analysis

The pairwise meta-analysis was performed using the STATA software version 14 (STATA Corporation, College Station, Texas). We performed a Bayesian network meta-analysis using the STATA software version 14 (STATA Corporation, College Station, Texas) and the package “rjags” (version 4-12, https://CRAN.R-project.org/package=rjags) plus the package “GeMTC” (version 1.0-1, https://CRAN.R-project.org/package=gemtc) in R software version 4.0.2 (https://www.R-project.org). We mainly used R software to generate some graphs (i.e., trace plot, density plot, ranking probability plot, and node-splitting plot). The results were analyzed by intention-to-treat analysis.

In pairwise meta-analysis, risk ratios (RRs) were conducted. *I*^2^ was used to represent levels of heterogeneity, low heterogeneity was indicated by *I*^2^ < 25%, medium heterogeneity by *I*^2^ of 25–50%, and high heterogeneity by *I*^2^ > 50%. If heterogeneity existed, a random effect model was used for meta-analysis, otherwise, a fixed-effect model was chosen. We also did meta-regression to find the source of heterogeneity. Publication bias was assessed by visual inspection of funnel plots and by Egger's test. All tests were two-tailed, 95% confidence intervals (CI) were calculated, and *p*-values < 0.05 were considered statistically significant. We also carried out sensitivity analyses by removing the following types of studies from the primary outcome (i.e., MACE) analyses: (1) excluding trials with relatively small sample size (i.e., trials with included patients ≤ 100 in each group); (2) excluding older trials (i.e., trials before 2014); (3) excluding each study successively to test the stability of the results.

In network meta-analysis, we conducted odds ratios (ORs). We used random-effect model to account for heterogeneity between studies. Trace plot is used to diagnose the degree of convergence of the model. The node-splitting method was used to analyze local inconsistency. After the comparison of various interventions, the ranking probability plot was used to rank the advantages and disadvantages of the interventions.

## Results

### Search results

**Figure 1:**
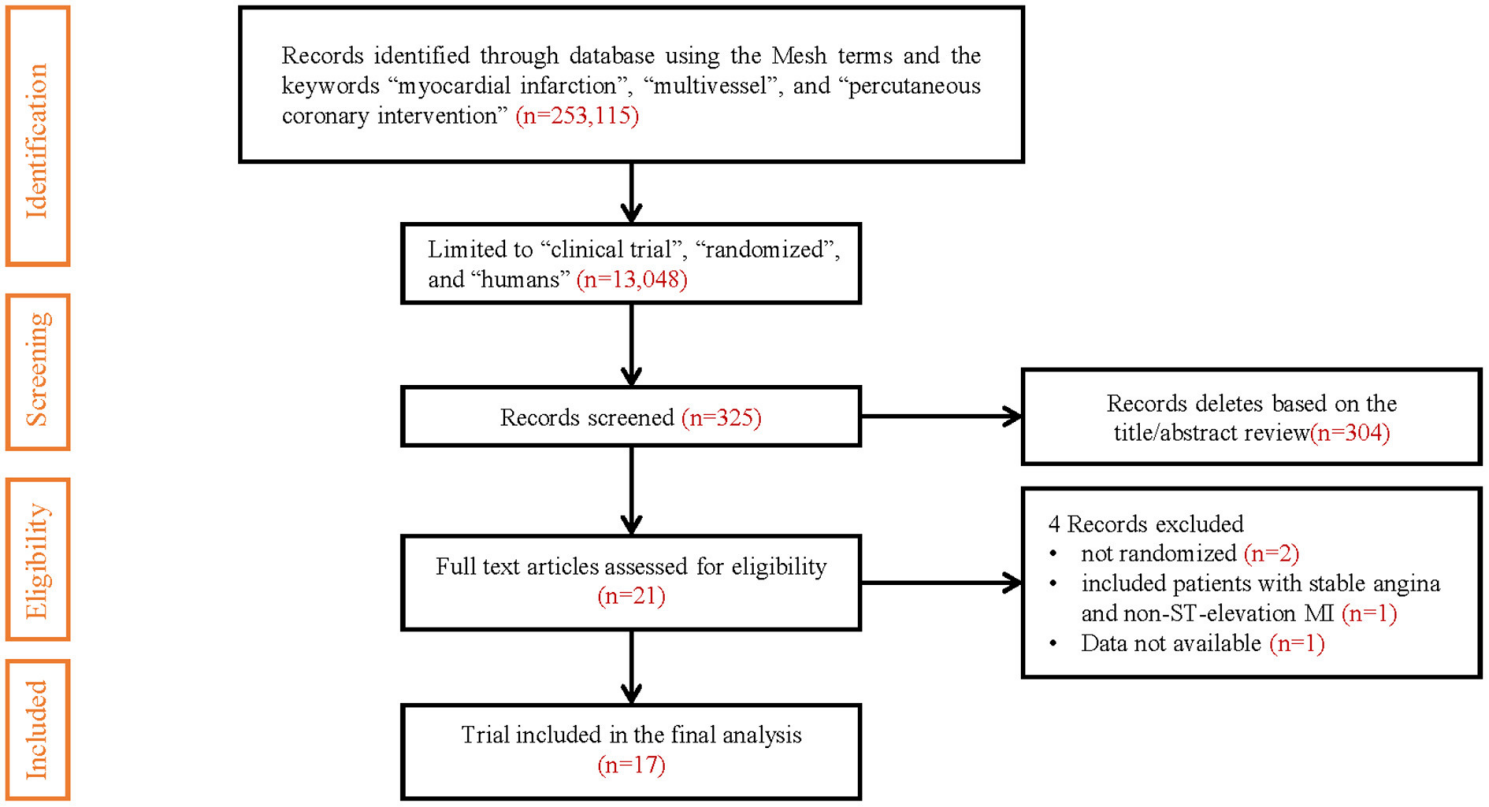
PRISMA study search flow diagram.

Of the 13,048 articles identified in the initial electronic search, 325 studies were preliminarily screened as relevant after reading titles and/or abstracts. Three studies reported both short- and long-term follow-up results, and we only included long-term follow-up results (7–9). Two studies comparing staged revascularization and complete revascularization at the index procedure were excluded because they weren't really randomized (10, 11). A randomized controlled trial comparing multivessel PCI and culprit-only revascularization that included patients with stable angina and non-ST-elevation MI was excluded (12). Another randomized controlled trial comparing immediate and staged complete revascularization, which reported only MACE event rates and all-cause mortality, did not report the number of specific events and the results of other outcome measures, was also excluded (4). The result of the search strategy is summarized in Figure 1.

### Characteristics of included studies

**Figure 2:**
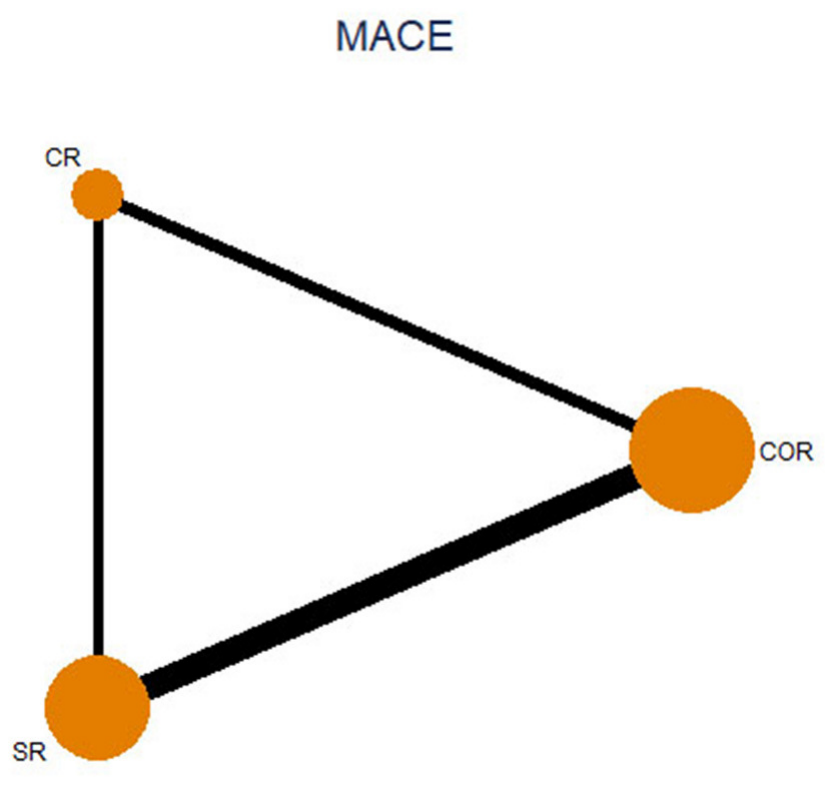
Network plot for major adverse cardiovascular event. CR, complete revascularization at the time of primary PCI; SR, complete revascularization as a staged procedure; COR, culprit-only revascularization.

17 studies with a total of 8,568 patients were included. There were 926 patients in the CR group, 3,415 in the SR group, and 4,227 in the COR group. The follow-up months ranged from 6 to 67.2 months (median 24 months). In our study, patients with cardiogenic shock were excluded. Three studies only compared the staged procedure and complete revascularization at the index procedure, so these three studies were not included in pairwise meta-analyses (5, 13, 14). Three studies compared three revascularization strategies (15–17). All characteristics of the included studies are summarized in Table 1. Most included studies were of high quality, Supplementary Table 3 and Supplementary Figure 1 summarizes the assessments of study quality. The network plot for the primary outcome is shown in Figure 2.

**Table 1:** Baseline characteristics of included trials.

**Trial/author**	**Year**	**Follow-up (median, months)**	**Comparison**	**Type of complete revascularization**	**Patient number**			**Timing of staged-procedure (Days)**	**The stenosis of non-culprit vessel lesion**
					**CR**	**SR**	**COR**		
PRIMA (13)	2004	12	CR vs. SR	Index and staged	37	35	NA	27.3^a^	≥70%
HLEP AMI (18)	2004	12	CR vs. COR	Index only	52	NA	17	NA	≥50%
Politi et al. (15)	2010	30	CR vs. SR vs. COR	Index and staged	65	65	84	NA	≥70%
Ghani et al. (7)	2012	36	SR vs. COR	Staged only	NA	80	41	7.5^b^	≥50%
PRAMI (19)	2013	23	CR vs. COR	Index only	234	NA	231	NA	≥50%
Prague 13 (20)	2015	38	SR vs. COR	Staged only	NA	106	108	3–40^c^	≥70%
CROSS-AMI (21)	2019	31	SR vs. COR	Staged only	NA	154	152	Hospitalization	visually assessed angiographic diameter stenosis ≥70% or a quantitative coronary angiography assessed diameter stenosis ≥50%
COMPLETE (3)	2019	36	SR vs. COR	Staged only	NA	2016	2025	1 (IQR, 1 to 3) or during the index hospitalization and 23 days (IQR, 12.5 to 33.5) or after hospital discharge	≥70%
CvLPRIT (16)	2019	67.2	CR vs. SR vs. COR	Index and staged	97	42	146	Hospitalization	≥70%
Compare-acute (8)	2020	36	CR vs. COR	Index only	295	NA	590	2.1 ± 1.0^a^	≥50%
DANAMI-3-PRIMULTI (22)	2015	27	SR vs. COR	Staged only	NA	314	313	2 (IQR 2 to 4)	≥50%
Estevez Loureiro et al. (23)	2014	12	SR vs. COR	Staged only	NA	100	99	NA	NA
Zhang et al. (24)	2015	24	SR vs. COR	Staged only	NA	215	213	7–10^c^	75%-90%
Hamza et al. (17)	2016	6	CR vs. SR vs. COR	Index and staged	NA	50	50	3	≥70%
Zhao et al. (25)	2016	12	SR vs. COR	Staged only	NA	148	158	7–10^c^	≥70%
Tarasov et al. (14)	2017	12	CR vs. SR	Index and staged	67	69	NA	10.1 ± 5.1^a^	≥70%
Mihnea et al. (5)	2021	12	CR vs. SR	Index and staged	50	50	NA	4.4^a^	≥75%

Summary of the characteristics of the included studies. NA, not applicable; IQR, interquartile range; CR, complete revascularization at the time of primary PCI; SR, complete revascularization as a staged procedure; COR, culprit-only revascularization; HELP-AMI, HEpacoat for cuLPrit or multivessel stenting for acute myocardial infarction; CROSS-AMI, complete revascularization or streSS echo in patients with multivessel disease and ST-segment elevation acute myocardial infarction; Compare-Acute, comparison between FFR guided revascularization vs. conventional strategy in acute STEMI patients with MVD; COMPLETE, complete vs. culprit-only revascularization to treat multi-vessel disease after early PCI for STEMI; CvLPRIT, complete vs. lesion-only PRimary PCI pilot study; DANAMI-3-PRIMULTI, third danish study of primary PCI in patients with ST-elevation myocardial infarction and multivessel disease: treatment of culprit lesion only or complete revascularization; PRAMI, preventive angioplasty in acute myocardial infarction. ^*a*^Mean. ^*b*^Median. ^*c*^Range.

In CvLPRIT (Complete vs. Lesion-only Primary percutaneous coronary Intervention Trial) (16), 97 patients were assigned to the CR group and 42 to the SR group. However, in the 12-month follow-up results reported in 2015, only the incidence of MACE events was reported for comparison between the CR and SR groups. In the 67.2-month follow-up results reported in 2019, data were not available to compare outcomes between the CR and SR groups. As we included the long-term follow-up results of the CvLPRIT trial in 2019, we excluded the CvLPRIT trial in our network meta-analysis. In the pairwise meta-analysis, we included all the follow-up results of the CvLPRIT trial. In HAMZA 2016 (17), only the number of patients in the CR and SR groups was reported, but no follow-up results were reported for either group. We have therefore not included this trial in our network meta-analysis.

### Pairwise meta-analysis

**Figure 3:**
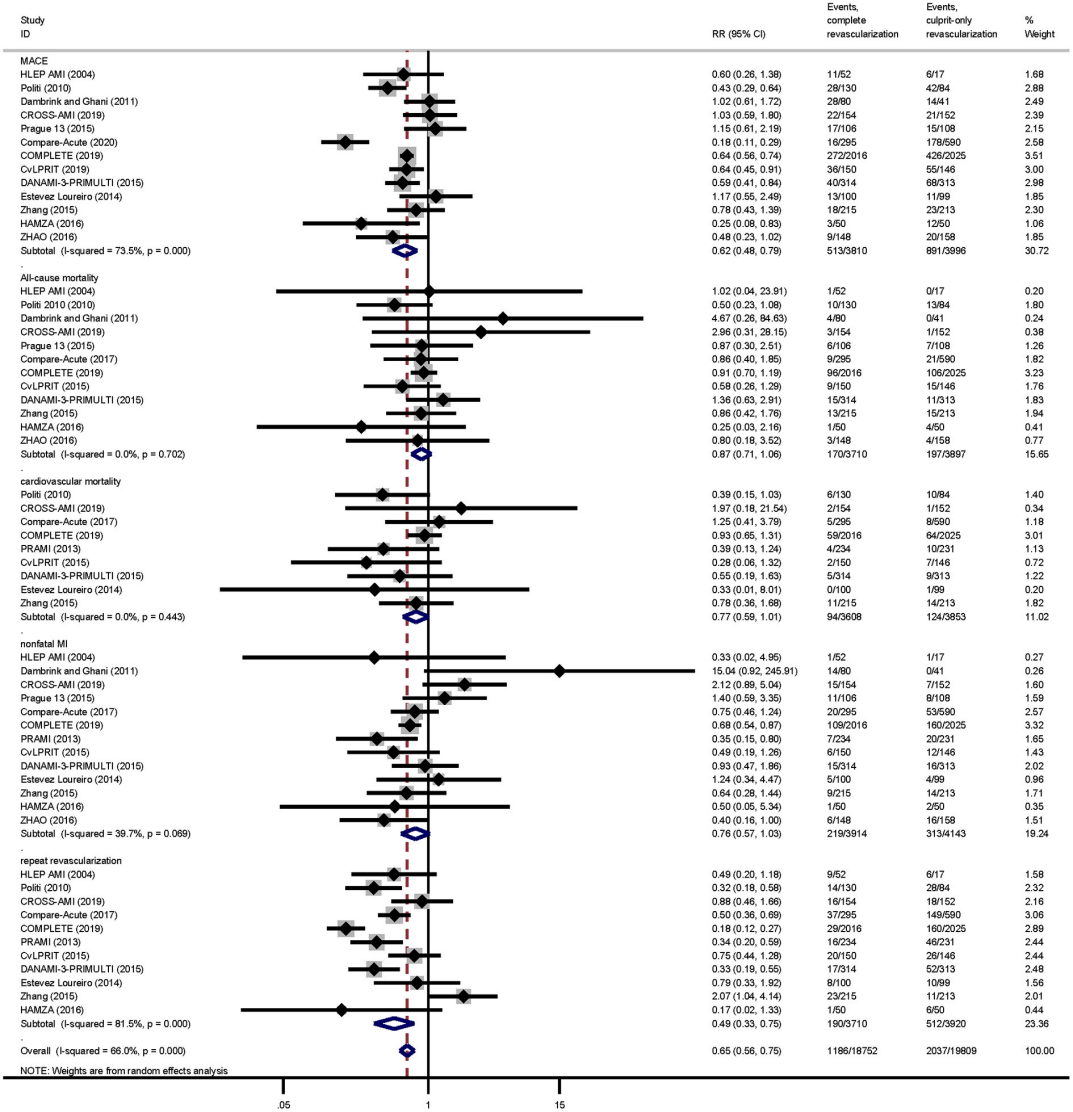
Pairwise meta-analysis main outcomes. Summary forest plot for major adverse cardiac events, cardiovascular mortality, all-cause mortality, repeat revascularization, and nonfatal myocardial infarction in the pairwise meta-analysis. CI, confidence interval; MACE, major adverse cardiac event(s); RR, risk ratio; MI, myocardial infarction; Other abbreviations as in Table 1.

Complete revascularization (i.e., either staged revascularization or complete revascularization at the index procedure) can reduce the incidence of MACE events compared to culprit-only revascularization (13.5 vs. 22.3%, RR: 0.62; 95% CI: 0.48–0.79; *p* < 0.001; *I*^2^ = 73.5%). This is mainly due to the reduced risk of repeat revascularization (5.1 vs. 13.1%, RR: 0.49; 95% CI: 0.33–0.75; *p* = 0.001; *I*^2^ = 81.5%). There were no significant differences in all-cause mortality (4.6 vs. 5.1%, RR: 0.87; 95% CI: 0.71–1.06; *p* = 0.169; *I*^2^ = 0.0%), cardiovascular mortality (2.6 vs. 3.2%, RR: 0.77; 95% CI: 0.59–1.01; *p* = 0.060; *I*^2^ = 0.0%), or risk of nonfatal MI (5.6 vs. 7.6%, RR: 0.76; 95% CI: 0.57–1.03; *p* = 0.074; *I*^2^ = 39.7%; Figure 3) between the two groups.

**Figure 4:**
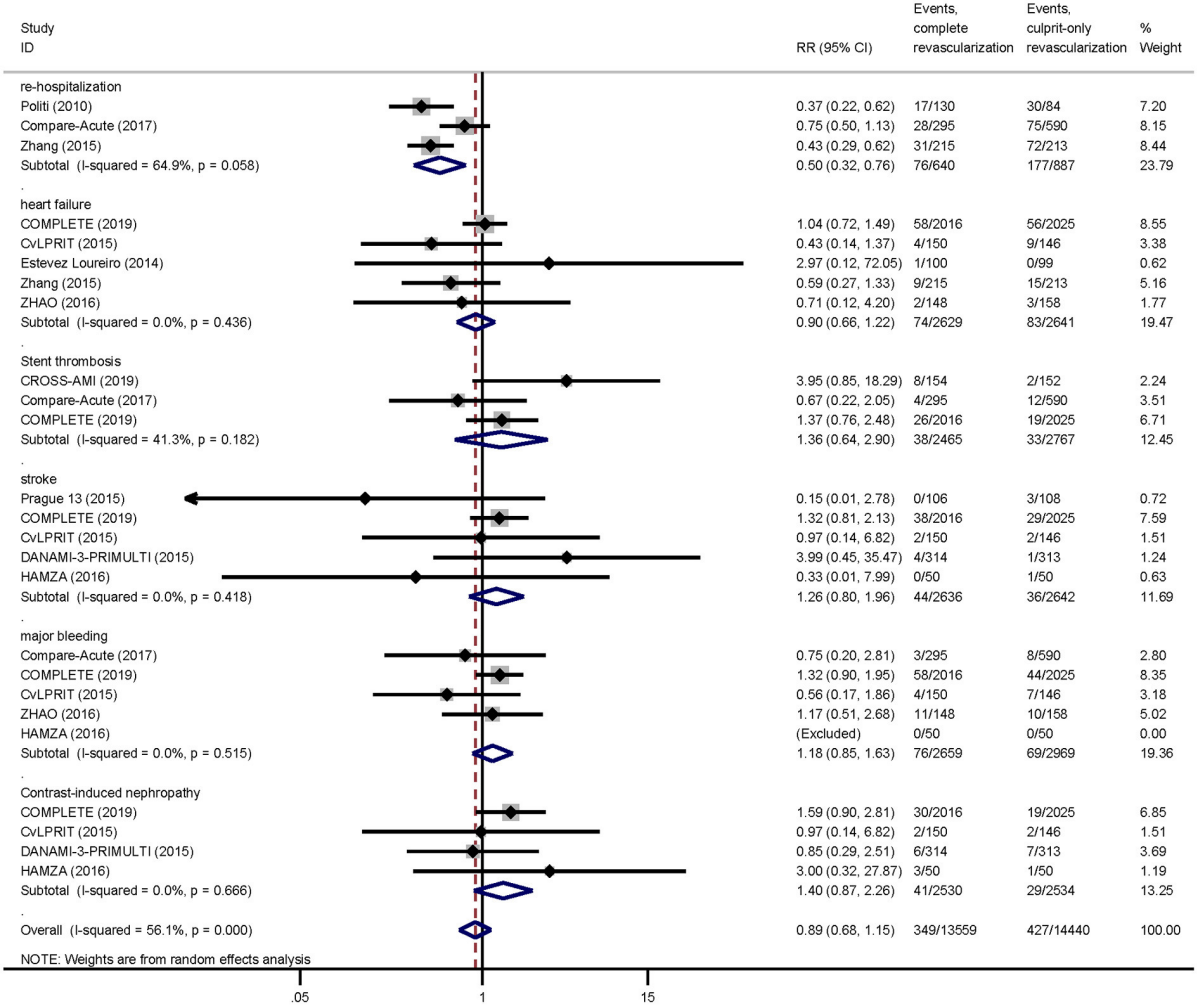
Pairwise meta-analysis other outcomes. Summary forest plot for re-hospitalization, heart failure, stent thrombosis, stroke, major bleeding, contrast-induced nephropathy. CI, confidence interval; MACE, major adverse cardiac event(s); RR, risk ratio; MI, myocardial infarction; Other abbreviations as in Table 1.

As for safety outcomes, the risk of re-hospitalization was lower in the complete revascularization group than in the culprit-only revascularization group (11.9 vs. 20.0%, RR: 0.50; 95% CI: 0.32–0.76; *p* = 0.001; *I*^2^ = 64.9%). And there was no significant difference between the two groups in the risk of heart failure (2.8 vs. 3.1%, RR: 0.90; 95% CI: 0.66–1.22; *p* = 0.491; *I*^2^ = 0.0%), stent thrombosis (1.5 vs. 1.2%, RR: 1.36; 95% CI: 0.64–2.90; *p* = 0.428; *I*^2^ = 41.3%), stroke (1.7 vs. 1.4%, RR: 1.26; 95% CI: 0.80–1.96; *p* = 0.316; *I*^2^ = 0.0%), major bleeding (2.9 vs. 2.3%, RR: 1.18; 95% CI: 0.85–1.63; *p* = 0.324; *I*^2^ = 0.0%), or contrast-induced nephropathy (1.6 vs. 1.1%, RR: 1.40; 95% CI: 0.87–2.26; *p* = 0.163; *I*^2^ = 0.0%; Figure 4).

Meta-regression analysis showed that follow-up months was not a source of heterogeneity in any results (Supplementary Figure 2). There was no publication bias for any of the outcomes based on Egger's test (Supplementary Table 4). The outcomes of the pairwise meta-analysis were summarized in Table 2, and the RR value and its 95% CI of each outcome are summarized in Supplementary Figure 3.

**Table 2:** Summary for the outcomes of the pairwise meta-analysis.

**Outcome**	**Incidence CR + SR/COR, %/%**	**RR**	**95% CI**	***p*-value**	***I*^2^, %**
MACE	13.5/22.3	0.62	0.48–0.79	< 0.001	73.5
All cause mortality	4.6/5.1	0.87	0.71–1.06	0.169	0.0
Cardiac mortality	2.6/3.2	0.77	0.59–1.01	0.060	0.0
Nonfatal MI	5.6/7.6	0.76	0.57–1.03	0.074	39.7
Repeat revascularization	5.1/13.1	0.49	0.33–0.75	0.001	81.5
Re-hospitalization	11.9/20.0	0.50	0.32–0.76	0.001	64.9
Heart failure	2.8/3.1	0.90	0.66–1.22	0.491	0.0
Stent thrombosis	1.5/1.2	1.36	0.64–2.90	0.428	41.3
Stroke	1.7/1.4	1.26	0.80–1.96	0.316	0.0
Major bleeding	2.9/2.3	1.18	0.85–1.63	0.324	0.0
Contrast-induced nephropathy	1.6/1.1	1.40	0.87–2.26	0.163	0.0

RR, risk ratio; MACE, major adverse cardiac event (s); CI, confidence interval; MI, myocardial infarction; CR, complete revascularization at the time of primary PCI; SR, complete revascularization as a staged procedure; COR, culprit-only revascularization.

For the primary outcome MACE, we conducted sensitivity analysis from the following aspects: (1) excluding trials with relatively small sample size (i.e., trials with included patients ≤ 100 in each group; RR: 0.60; 95% CI: 0.44–0.83; *p* = 0.002); (2) excluding some older trials (i.e., trials before 2014; RR: 0.61; 95% CI: 0.45–0.82; *p* = 0.001); (3) excluding each study successively, there was no significant change in the results (Supplementary Figure 4).

### Network meta-analysis

**Figure 5:**
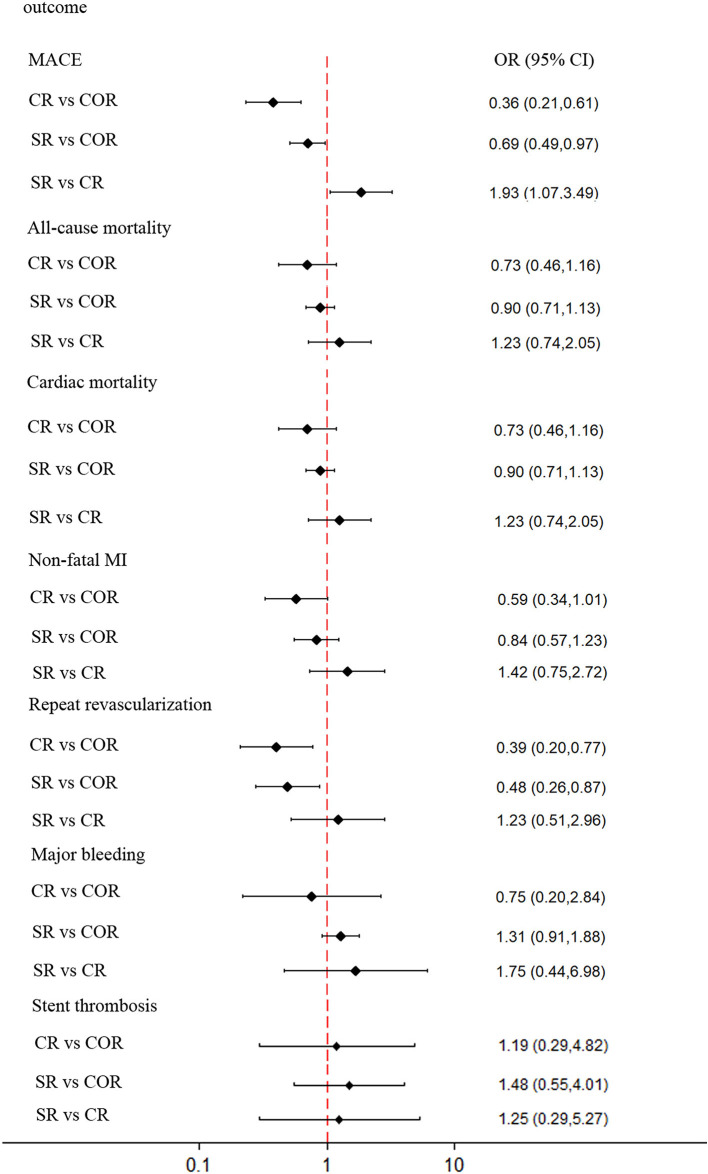
Network meta-analysis outcomes. Forest plot for the network meta-analysis comparing the 3 different revascularization strategies for MACE, all-cause mortality, cardiovascular mortality, nonfatal myocardial infarction, repeat revascularization, major bleeding, and stent thrombosis. Diamonds represent OR values, and horizontal lines represent 95% confidence intervals. MACE, major adverse cardiac event (s); MI, myocardial infarction; OR, odds ratio; CR, complete revascularization at the time of primary PCI; SR, complete revascularization as a staged procedure; COR, culprit-only revascularization.

**Figure 6:**
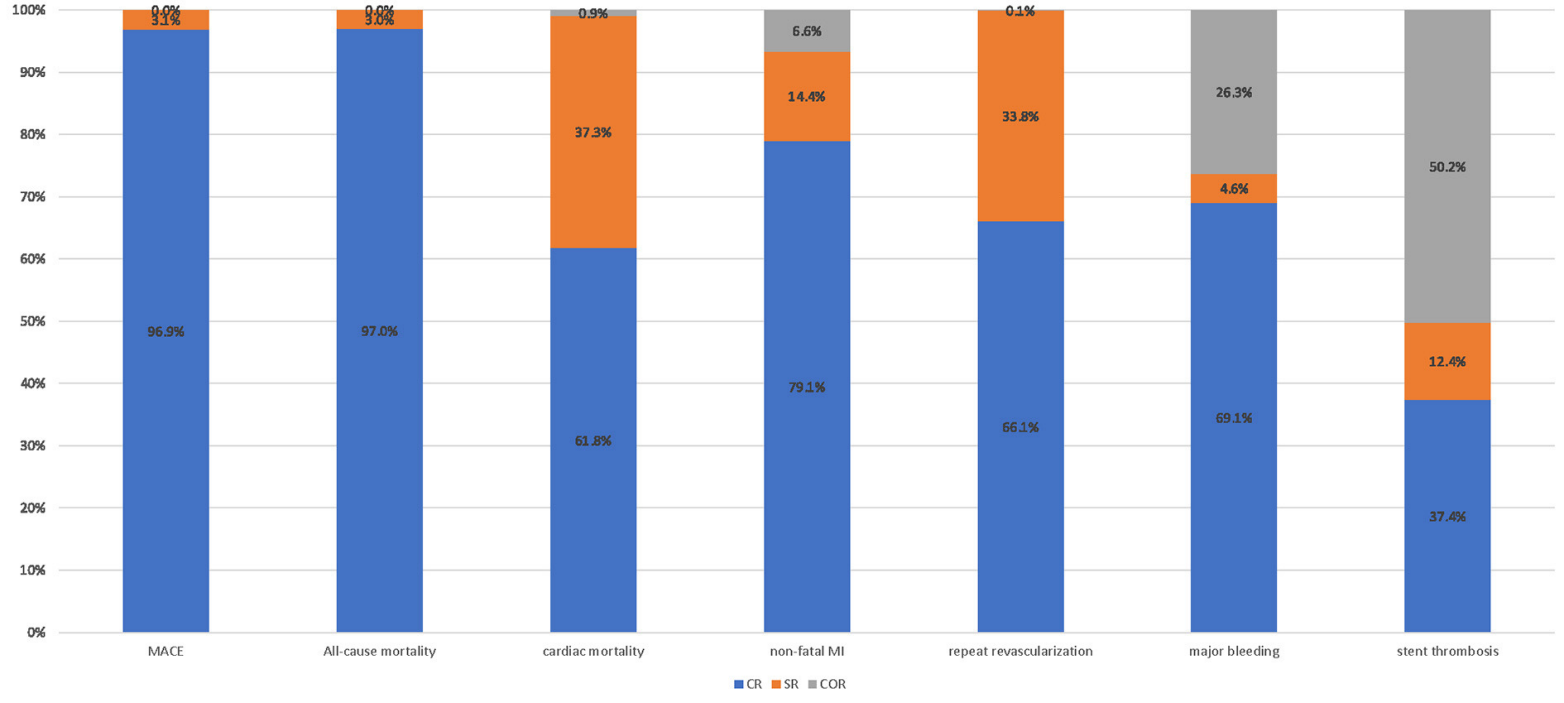
Probability ranking plot of each strategy. Probability ranking plot of three revascularization strategies. MACE, major adverse cardiac event (s); MI, myocardial infarction; OR, odds ratio; CR, complete revascularization at the time of primary PCI; SR, complete revascularization as a staged procedure; COR, culprit-only revascularization.

For network meta-analysis, complete revascularization as a staged procedure (OR: 0.69; 95% CI: 0.49–0.97; Figure 5), and at the index procedure (OR: 0.36; 95% CI: 0.21–0.61) can reduce the risk of MACE compared with culprit-only revascularization, respectively. However, staged revascularization (OR: 1.93; 95% CI: 1.07–3.49) will increase the risk of MACE compared with the index procedure. Staged revascularization (OR: 0.48; 95% CI: 0.26–0.87) and Complete revascularization at the index procedure (OR: 0.39; 95% CI: 0.20–0.77) were associated with a lower risk of repeat revascularization compared with culprit-only revascularization, but there was no significant difference between the two groups (OR: 1.23; 95% CI: 0.51–2.96). There were no significant differences among the three revascularization strategies in the risk of all-cause mortality, cardiac mortality, non-fatal MI, major bleeding, or stent thrombosis. A network meta-analysis for the outcome of re-hospitalization, contrast-induced nephropathy, heart failure, and stroke was not performed because there were not enough studies (i.e., there were only three studies of re-hospitalization, two studies of contrast-induced nephropathy, four studies of heart failure, three studies of stroke). The probability ranking plot of each strategy is shown in Figure 6. For the outcome of MACE, CR was associated with the highest likelihood of optimal strategy (96.9%), followed by SR (3.1%) and COR (0.0%). Meanwhile, CR was associated with the highest likelihood of optimal strategy in terms of all-cause mortality (97.0%), cardiac mortality (61.8%), non-fatal MI (79.1%), repeat revascularization (66.1%), major bleeding (69.1%). However, COR was associated with the highest likelihood of optimal strategy in terms of stent thrombosis (50.2%). All clinical outcome analysis models can achieve a good degree of convergence, and we estimated the convergence of model parameters by visually inspecting trace and density plots (Supplementary Figure 5). Local inconsistency models were not statistically significant for all outcomes (Supplementary Figure 6).

## Discussion

A total of 17 studies containing 8,568 patients were included in our study. There were 926 patients in the CR group, 3,415 in the SR group, and 4,227 in the COR group. Our objective was to compare the effects of three revascularization strategies on the outcomes of STEMI patients with multivessel coronary disease. The final results suggest that there is no significant difference in the risk of all-cause mortality, cardiac mortality, non-fatal MI, major bleeding, or stent thrombosis among the three revascularization strategies at the median of 24 months. This result also implied that revascularization of non-culprit vessels during PPCI is safe and feasible while reducing the risk of re-hospitalization. Both CR and SR were associated with a reduced risk of MACE events compared with culprit-only revascularization. It is important to highlight that this result was driven only by repeat revascularization. It is worth mentioning that a sub-study of the COMPLETE trial suggested that, among STEMI patients with multi-vessel disease, the benefit of complete revascularization over culprit-lesion only PCI was consistent irrespective of the investigator-determined timing of non-culprit-lesion intervention (26). This is also consistent with our findings. Our analysis also found that SR was associated with an increased risk of MACE events compared with CR.

The additional benefits of CR or SR were not supported in previous meta-analyses (27–30). The following reasons may help to explain this evidence gap. First, there are potential confounding factors, such as types of studies included, the timing of the treatment of the non-culprit arteries, the severity of the disease (i.e., patients were hemodynamically stable or with cardiogenic shock), duration of follow-up months, definitions for measuring clinical outcome, patient inclusion criteria, and statistical methods. Second, the sample size of the previous meta-analysis was not sufficient to test some outcomes. A previous meta-analysis based on RRs and event rate in the culprit-only group suggested that more than 8,000 patients would be required to achieve a test efficacy of 80% for all-cause mortality (31). To avoid these limitations of the previous meta-analysis, only RCTs published in peer-reviewed journals were included and high-risk patients were excluded (i.e., patients with cardiogenic shock), while confounding factors were analyzed. Sensitivity analysis was performed for the duration of follow-up. On the other hand, we included the COMPLETE trial and some other RCTs (3, 5), increasing the sample size of our meta-analysis to 8,568 patients.

Another troubling question is the optimal timing of PCI for non-culprit vessels. Some studies have discussed a reduced or similar risk of adverse outcomes if complete revascularization is performed in non-culprit lesions at the time of primary PCI (32, 33), while other studies have shown that planned staged complete revascularization of non-culprit lesions is more effective (15, 34, 35). In response to this problem, we conducted a network meta-analysis among these three revascularization strategies. In addition, a recent study suggested that early or delayed revascularization had no significant impact on clinic outcomes (36). This may be due to some confounding factors such as the research method and grouping method. In this meta-analysis, the authors classified SR < 7 days and CR into the early revascularization group and classified SR >7 days as the delayed revascularization group.

Our study has some limitations. First, not all included trials assessed safety outcomes, which resulted in some safety outcomes not being analyzed in our network meta-analysis due to an insufficient number of studies. Second, many studies excluded patients with previous CABG, cardiogenic shock, complex lesions such as left main disease, or chronic total occlusion, therefore the results of this meta-analysis should not be extrapolated in these patients. Third, various studies have different definitions for multivessel coronary disease and MACE events. For example, some studies defined multivessel coronary disease as more than one non-culprit artery stenosis diameter >50% confirmed by angiography (7, 8, 18, 19, 22), while the other studies defined multivessel coronary disease as stenosis diameter >70% in more than one non-culprit artery (3, 9, 15, 17). Furthermore, our meta-analysis was unable to stratify outcomes according to the severity or complexity of non-culprit arteries due to the reason that this is a research-level meta-analysis, which means we cannot make certain adjustments to the outcomes for certain patient characteristics that may have affected the results of the study. In the subgroup analysis of the COMPLETE trial (3), for non-culprit lesion with stenosis of 80% on visual estimation or 60% on laboratory assessment, regardless of when complete revascularization was performed, was associated with greater reductions in the risk of first coprimary outcome (composite of death from cardiovascular causes or new myocardial infarction) and second coprimary outcome (composite of death from cardiovascular causes, new myocardial infarction, or ischemia-driven revascularization). This suggests that the severity of non-culprit lesion may be the key to determining the timing of PCI in non-culprit lesions. These may explain the heterogeneity among studies. Finally, there are few studies directly comparing CR and SR in our meta-analysis. The ongoing BIOVASC trial (37) and the MULTISTARS AMI trial (38), and the OPTION-STEMI trial (39), which directly compare CR and SR, will further elucidate the impact of the three revascularization strategies on clinical outcomes.

There is still much debate about the management of non-culprit lesions in patients with multivessel coronary artery disease. For example, for non-culprit intermediate coronary stenosis (50–70% diameter stenosis), physiological assessments [such as fractional flow reserve (FFR), instantaneous wave-free ratio (iFR)] and intravascular imaging [such as Optical coherence tomography (OCT)] may be required to determine the optimal timing of intervention of non-culprit lesions (40). Some meta-analyses and RCTs have compared FFR-guided vs. coronary angiography-guided complete revascularization on clinical outcomes (41–45). More research is needed in the future to provide more evidence for optimal timing of revascularization for non-culprit lesions in patients with multivessel coronary artery disease.

## Conclusion

Our analysis suggests that complete revascularization should be performed in STEMI patients with multivessel coronary artery disease, and complete revascularization at the index procedure is superior to complete revascularization as a staged procedure in reducing the risk of MACE events. More future RCTs are needed to determine the effect of complete revascularization on all-cause mortality, cardiovascular mortality, and risk of re-MI, as well as the optimal timing of complete revascularization.

## Data availability statement

The original contributions presented in the study are included in the article/Supplementary material, further inquiries can be directed to the corresponding authors.

## Author contributions

YF: conceptualization, methodology, software, data curation, formal analysis, writing—original draft, and visualization. SL: validation, data curation, and formal analysis. SH: data curation, and visualization. JW: supervision, and writing—review and editing. HS: supervision, and writing—review and editing. All authors have read and approved the final manuscript.

## Funding

This study was supported by the Innovation and Technology Fund of Zhongnan Hospital, Wuhan University (Grant number cxpy20160027) and Detection and prevention of dilated cardiomyopathy evolving from viral myositis (Grant number 212000028).

## Conflict of interest

All authors declare that they have no known competing financial interests or personal relationships that could have appeared to influence the work reported in this paper.

## Publisher's note

All claims expressed in this article are solely those of the authors and do not necessarily represent those of their affiliated organizations, or those of the publisher, the editors and the reviewers. Any product that may be evaluated in this article, or claim that may be made by its manufacturer, is not guaranteed or endorsed by the publisher.

## References

[1] Park DW Clare RM Schulte PJ Pieper KS Shaw LK Califf RM . Extent, location, and clinical significance of non-infarct-related coronary artery disease among patients with ST-elevation myocardial infarction. JAMA. (2014) 312:2019–27. 10.1001/jama.2014.15095 25399277

[2] Levine GN Bates ER Blankenship JC Bailey SR Bittl JA Cercek B . 2015 ACC/AHA/SCAI focused update on primary percutaneous coronary intervention for patients with ST-elevation myocardial infarction: an update of the 2011 ACCF/AHA/SCAI guideline for percutaneous coronary intervention and the 2013 ACCF/AHA guideline for the management of ST-elevation myocardial infarction: a report of the American college of cardiology/American heart association task force on clinical practice guidelines and the society for cardiovascular angiography and interventions. Circulation. (2016) 133:1135–47. 10.1161/CIR.0000000000000336 26490017

[3] Mehta SR Wood DA Storey RF Mehran R Bainey KR Nguyen H . Complete revascularization with multivessel PCI for myocardial infarction. N Engl J Med. (2019) 381:1411–21. 10.1056/NEJMoa1907775 31475795

[4] Park S Choi B Choi S Byun J Cha J Lee K . Abstract 12825: immediate vs. staged complete revascularization in acute ST-segment elevation myocardial infarction patients with multi-vessel disease undergoing primary percutaneous coronary intervention: prospective, randomized, multicenter trial of complete vs. culprit lesion revascularization in acute ST-segment elevation myocardial infarction patients with multivessel disease undergoing primary percutaneous coronary intervention with everolimus eluting stent (cocua Trial). Circulation. (2021) 144(Suppl_1):A12825. 10.1161/circ.144.suppl_1.1282526078378

[5] Brendea M Popescu MI Popa V Carmen P. A clinical trial comparing complete revascularization at the time of primary percutaneous coronary intervention vs. during the index hospital admission in patients with multi-vessel coronary artery disease and STEMI uncomplicated by cardiogenic shock. Anatol J Cardiol. (2021) 25:781–8. 10.5152/AnatolJCardiol.2021.71080 34734811 PMC8575400

[6] Hutton B Salanti G Caldwell DM Chaimani A Schmid CH Cameron C . The PRISMA extension statement for reporting of systematic reviews incorporating network meta-analyses of health care interventions: checklist and explanations. Ann Intern Med. (2015) 162:777–84. 10.7326/M14-2385 26030634

[7] Ghani A Dambrink JE van Hof AWJ Ottervanger JP Gosselink ATM Hoorntje JCA. Treatment of non-culprit lesions detected during primary PCI: long-term follow-up of a randomised clinical trial. Netherlands Heart J. (2012) 20:347–53. 10.1007/s12471-012-0281-y 22622701 PMC3430767

[8] Smits PC Laforgia PL Abdel-Wahab M Neumann F Richardt G Boxma-de Klerk B . Fractional flow reserve-guided multivessel angioplasty in myocardial infarction: 3-year follow-up with cost benefit analysis of the Compare-Acute trial. EuroInterv J EuroPCR Collaborat Work Group Intervent Cardiol Eur Soc Cardiol. (2020) 16:225–32. 10.4244/EIJ-D-20-00012 32250250

[9] Gershlick AH Banning AS Parker E Wang D Budgeon CA Kelly DJ . Long-term follow-up of complete vs. lesion-only revascularization in STEMI and multivessel disease: the CvLPRIT trial. J Am Coll Cardiol. (2019) 74:3083–94. 10.1016/j.jacc.2019.10.033 31856964

[10] Saad M Rashed A. El-kilany W, El-Haddad M, Elgendy IY. Preliminary report on the safety and efficacy of staged vs complete revascularization in patients with multivessel disease at the time of primary percutaneous coronary intervention. Int J Angiol. (2017) 26:143–7. 10.1055/s-0036-1572522 28804231 PMC5552895

[11] Maamoun W Elkhaeat N Elarasy R. Safety and feasibility of complete simultaneous revascularization during primary PCI in patients with STEMI and multi-vessel disease. Egy Heart J. (2011) 63:39–43. 10.1016/j.ehj.2011.08.030

[12] Fagel ND Maarse M Slagboom T Herrman JP Van Der Schaaf RJ Amoroso G . 5-year results of the complete vs. culprit vessel percutaneous coronary intervention in multivessel disease using drug-eluting stents II study a prospective, randomized controlled trial. Eur Heart J. (2017) 38:270. 10.1093/eurheartj/ehx502.P1374 PMC6533316 30868547

[13] Ochala A Smolka GA Wojakowski W Dudek D Dziewierz A Krolikowski Z . The function of the left ventricle after complete multivessel one-stage percutaneous coronary intervention in patients with acute myocardial infarction. J Invasive Cardiol. (2004) 16:699–702. 15596873

[14] Tarasov RS Ganyukov VI Barbarash OL Barbarash LS. Two preventive multivessel stenting strategy with zotarolimus eluting stents in STelevation myocardial infarction patients: 12-month results of randomized trial. Intervent Cardiol. (2017) 9:57–63. 10.4172/Interventional-Cardiology.1000555

[15] Politi L Sgura F Rossi R Monopoli D Guerri E Leuzzi C . A randomised trial of target-vessel vs. multi-vessel revascularisation in ST-elevation myocardial infarction: major adverse cardiac events during long-term follow-up. Heart. (2010) 96:662–7. 10.1136/hrt.2009.177162 19778920

[16] Gershlick AH Banning AS Parker E Wang D Budgeon CA Kelly DJ . Complete vs. lesion-only revascularization in patients with STEMI and multivessel disease: the CvLPRIT trial. J Am Coll Cardiol. (2019) 74:3083–94. 31856964 10.1016/j.jacc.2019.10.033

[17] Hamza M Elgendy I. A randomized trial of complete vs. culprit-only revascularization during primary percutaneous coronary intervention in diabetic patients with acute ST elevation myocardial infarction and multi vessel disease. J Am Coll Cardiol. (2016) 68:B56–7. 10.1016/j.jacc.2016.09.281 27245121

[18] Di Mario C Mara S Flavio A Imad S Antonio M Anna P . Single vs. multivessel treatment during primary angioplasty: results of the multicentre randomised HEpacoat for cuLPrit or multivessel stenting for acute myocardial infarction (HELP AMI) study. Int J Cardiovasc Intervent. (2004) 6:128–33. 10.1080/14628840310030441 16146905

[19] Wald DS Morris JK Wald NJ Chase AJ Edwards RJ Hughes LO . Randomized trial of preventive angioplasty in myocardial infarction. N Engl J Med. (2013) 369:1115–23. 10.1056/NEJMoa1305520 23991625

[20] Hlinomaz O Groch L Poloková K Lehar F Vekov T Petkov R . Multivessel coronary disease diagnosed at the time of primary PCI for STEMI: complete revascularisation vs. conservative strategy Prague-13 trial. Kardiologicka Revue. (2015) 17:214–20.

[21] Calviño-Santos R Estévez-Loureiro R Peteiro-Vázquez J Salgado-Fernández J Rodríguez-Vilela A Franco-Gutiérrez R . Angiographically guided complete revascularization vs. selective stress echocardiography-guided revascularization in patients with ST-segment-elevation myocardial infarction and multivessel disease: the CROSS-AMI randomized clinical trial. Circul Cardiovasc Intervent. (2019) 12:e007924. 10.1161/CIRCINTERVENTIONS.119.007924 31554422

[22] Engstrom T Kelbaek H Helqvist S Hofsten DE Klovgaard L Holmvang L . Complete revascularisation vs. treatment of the culprit lesion only in patients with ST-segment elevation myocardial infarction and multivessel disease (DANAMI-3–PRIMULTI): an open-label, randomised controlled trial. Lancet. (2015) 386:665–71. 10.1016/S0140-6736(15)60648-1 26347918

[23] Estevez Loureiro R Calvino-Santos R Peteiro J Bouzas-Mosquera A Salgado-Fernandez J Soler-Martin MR . Preventive revascularization does not offer clinical advantage over a selective invasive strategy in patients with ST-segment elevation myocardial infarction and multivessel disease. Eur Heart J. (2014) 35:477. 10.1093/eurheartj/ehu323

[24] Zhang J Wang Q Yang H Ma L Fu X Hou W . Evaluation of different revascularization strategies for patients with acute myocardial infarction with lesions of multiple coronary arteries after primary percutaneous coronary intervention and its economic evaluation. Chin Crit Care Med. (2015) 27:169–74. 10.3760/cma.j.issn.2095-4352.2015.03.003 25757964

[25] Zhao B Peng J Ren L Lei L Wang Z Ye H. Conservative pharmacotherapy vs. staged percutaneous coronary intervention for non-culprit vessels in patients with ST-segment elevation myocardial infarction. Exp Ther Med. (2016) 12:4147–53. 10.3892/etm.2016.3895 28105141 PMC5228445

[26] Wood DA Cairns JA Wang J Mehran R Storey RF Nguyen H . Timing of staged nonculprit artery revascularization in patients with ST-segment elevation myocardial infarction: complete trial. J Am Coll Cardiol. (2019) 74:2713–23. 10.1016/j.jacc.2019.09.051 31779786

[27] Bajraktari G Jashari H Ibrahimi P Alfonso F Jashari F Ndrepepa G . Complete revascularization for patients with ST-segment elevation myocardial infarction and multivessel coronary artery disease: a meta-analysis of randomized trials. Coron Artery Dis. (2018) 29:204–15. 10.1097/MCA.0000000000000602 29346126

[28] Witberg G Kornowski R. Current perspectives on revascularization in multivessel ST elevation myocardial infarction. Coron Artery Dis. (2017) 28:498–506. 10.1097/MCA.0000000000000496 28537939

[29] Gupta A Bajaj NS Arora P Arora G Qamar A Bhatt DL. FFR-guided multivessel stenting reduces urgent revascularization compared with infarct-related artery only stenting in ST-elevation myocardial infarction: a meta-analysis of randomized controlled trials. Int J Cardiol. (2018) 252:63–7. 10.1016/j.ijcard.2017.07.041 29249439 PMC5758864

[30] Bravo CA Hirji SA Bhatt DL Kataria R Faxon DP Ohman EM . Complete vs. culprit-only revascularisation in ST elevation myocardial infarction with multi-vessel disease. Cochrane Database Syst Rev. (2017) 5:CD011986. 10.1002/14651858.CD011986.pub2 28470696 PMC6481381

[31] Elgendy IY Mahmoud AN Kumbhani DJ Bhatt DL Bavry AA. Complete or culprit-only revascularization for patients with multivessel coronary artery disease undergoing percutaneous coronary intervention: a pairwise and network meta-analysis of randomized trials. JACC Cardiovasc Interv. (2017) 10:315–24. 10.1016/j.jcin.2016.11.047 28231899

[32] Bates ER Tamis-Holland JE Bittl JA O'Gara PT Levine GN PCI. strategies in patients with ST-segment elevation myocardial infarction and multivessel coronary artery disease. J Am Coll Cardiol. (2016) 68:1066–81. 10.1016/j.jacc.2016.05.086 27585512

[33] Pasceri V Patti G Pelliccia F Gaudio C Speciale G Mehran R . Complete revascularization during primary percutaneous coronary intervention reduces death and myocardial infarction in patients with multivessel disease: meta-analysis and meta-regression of randomized trials. JACC Cardiovasc Interv. (2018) 11:833–43. 10.1016/j.jcin.2018.02.028 29747913

[34] Neumann FJ Sousa-Uva M Ahlsson A Alfonso F Banning AP Benedetto U . 2018 ESC/EACTS guidelines on myocardial revascularization. Eur Heart J. (2019) 40:87–165. 10.1093/eurheartj/ehy855 30165437

[35] Li Z Zhou Y Xu Q Chen X. Staged vs. one-time complete revascularization with percutaneous coronary intervention in STEMI patients with multivessel disease: a systematic review and meta-analysis. PLoS ONE. (2017) 12:e169406. 10.1371/journal.pone.0169406 28107455 PMC5249143

[36] Abouelmagd K Tayel H Atta A Ladwiniec A Ibrahim M. Early vs. delayed complete revascularisation in patients presenting with ST-segment elevation myocardial infarction and multivessel disease: a systematic review and meta-analysis of randomised controlled trials. Open Heart. (2022) 9:e001975. 10.1136/openhrt-2022-001975 35728889 PMC9214375

[37] den Dekker WK Van Mieghem NM Bennett J Sabate M Esposito G van Bommel RJ . Percutaneous complete revascularization strategies using sirolimus-eluting biodegradable polymer-coated stents in patients presenting with acute coronary syndrome and multivessel disease: rationale and design of the BIOVASC trial. Am Heart J. (2020) 227:111–7. 10.1016/j.ahj.2020.06.006 32739537

[38] Stähli BE Varbella F Schwarz B Nordbeck P Felix SB Lang IM . Rationale and design of the MULTISTARS AMI trial: a randomized comparison of immediate vs. staged complete revascularization in patients with ST-segment elevation myocardial infarction and multivessel disease. Am Heart J. (2020) 228:98–108. 10.1016/j.ahj.2020.07.016 32871329

[39] NCT. Timing of FFR-Guided PCI for Non-IRA in STEMI and MVD (OPTION-STEMI) (2020). Available online at: https://clinicaltrials.gov/ct2/show/NCT04626882

[40] Montone RA Niccoli G Crea F Jang IK. Management of non-culprit coronary plaques in patients with acute coronary syndrome. Eur Heart J. (2020) 41:3579–86. 10.1093/eurheartj/ehaa481 32676644

[41] Elbadawi A Dang AT Hamed M Eid M Prakash HPM Saleh M . FFR- vs. angiography-guided revascularization for nonculprit stenosis in STEMI and multivessel disease: a network meta-analysis. JACC Cardiovasc Interv. (2022) 15:656–66. 10.1016/j.jcin.2022.01.002 35331458

[42] Puymirat E Cayla G Simon T Steg PG Montalescot G Durand-Zaleski I . Multivessel PCI guided by FFR or angiography for myocardial infarction. N Engl J Med. (2021) 385:297–308. 10.1056/NEJMoa2104650 33999545

[43] Piroth Z Omerovic E Klerk BBD Abdel-Wahab M Neumann FJ Richardt G . TCT-106 FFR in nonculprits in STEMI: a substudy of the COMPARE-ACUTE trial. J Am Coll Cardiol. (2019) 74:B106. 10.1016/j.jacc.2019.08.152

[44] NCT. FFR Driven Complete Revascularization Vs. Usual Care in NSTEMI Patients and Multivessel Disease (2018). Available online at: https://clinicaltrials.gov/ct2/show/NCT03562572

[45] Verardi R Fioravanti F Barbero U Conrotto F Omede P Montefusco A . Network meta-analysis comparing iFR vs. FFR vs. coronary angiography to drive coronary revascularization. J Interv Cardiol. (2018) 31:725–30. 10.1111/joic.12551 30136420

