# Supplementary material for: The optimal timing for non-culprit percutaneous coronary intervention in patients with multivessel coronary artery disease: A pairwise and network meta-analysis of randomized trials
Source: Front Cardiovasc Med. 2022 Sep 26;9:1000664. doi: 10.3389/fcvm.2022.1000664 (PMC9548605; doi:10.3389/fcvm.2022.1000664)

**Supplemental appendix contents:**

Supplemental Table 1: Search syntax of the electronic database

Supplemental Table 2: Definition of major outcomes

Supplemental Table 3: Summary of ROB 1.0 Assessment of included Studies

Supplemental Figure 1: Quality assessment of each study

Supplemental Figure 2: Meta-regression of follow-up months in two groups of revascularization

Supplemental Table 4: Summary of publication bias for all outcomes

Supplemental Figure 3: Pairwise meta-analysis summary of clinical outcomes

Supplemental Figure 4: Sensitivity analysis

Supplemental Figure 5: Trace and density plots of all outcomes

Supplemental Figure 6: Local inconsistency analysis of all outcomes

**Supplemental Table 1: Search syntax of the electronic database**

| Search Syntax of Medline – PubMed interface |
| --- |
| 1. "Myocardial Infarction"[MeSH Terms] 2. ("myocard*"[Title/Abstract] OR "heart"[Title/Abstract]) AND "infarct*"[Title/Abstract] |
| 1. ("cardiovas*"[Title/Abstract] OR "heart"[Title/Abstract]) AND "stroke*"[Title/Abstract] 2. "heart attack*"[Title/Abstract] 3. "cardiogenic shock"[Title/Abstract] 4. ("ST"[Title/Abstract] AND "elevat*"[Title/Abstract]) AND ("myocardial infarction*"[Title/Abstract] OR "MI"[Title/Abstract]) 5. "STEMI"[Title/Abstract] 6. #1 OR #2 OR #3 OR #4 OR #5 OR #6 OR #7 7. "Percutaneous Coronary Intervention"[MeSH Terms] 8. "percutaneous coronary"[Title/Abstract] AND ("intervention*"[Title/Abstract] OR "revascularization*"[Title/Abstract]) 9. "PCI"[Title/Abstract] OR "PPCI"[Title/Abstract] 10. (("transluminal"[Title/Abstract] OR "trans-luminal"[Title/Abstract]) OR "trans-luminal"[Title/Abstract]) AND "coronary"[Title/Abstract] 11. "Balloon"[Title/Abstract] AND ("coronary"[Title/Abstract] OR "dilat*"[Title/Abstract]) 12. "atherectom*"[Title/Abstract] 13. "angioplast*"[Title/Abstract] 14. "Stents"[MeSH Terms] 15. "stent*"[Title/Abstract] 16. #9 OR #10 OR #11 OR #12 OR #13 OR #14 OR #15 OR #16 OR #17 17. "multi*"[Title/Abstract] AND "Vessel"[Title/Abstract] 18. "Multivessel"[Title/Abstract] OR "multi-vessel"[Title/Abstract] OR "multi vessel"[Title/Abstract] 19. "infarct-related"[Title/Abstract] OR "infarct related"[Title/Abstract] OR "IRA"[Title/Abstract] OR "non infarct related"[Title/Abstract] OR "non-IRA"[Title/Abstract] 20. "culprit"[Title/Abstract] OR "culprit-only"[Title/Abstract] OR "non-culprit"[Title/Abstract] OR "nonculprit"[Title/Abstract] OR "bystander"[Title/Abstract] 21. #19 OR #20 OR #21 OR #22 22. "Clinical Trial"[Publication Type] 23. "randomized"[Title/Abstract] OR "placebo"[Title/Abstract] OR "randomly"[Title/Abstract] OR "trial"[Title] 24. #24 OR #25 25. "Animals"[MeSH Terms] 26. "Humans"[MeSH Terms] 27. #27 NOT (#27 AND #28) 28. #26 NOT #29 29. #8 AND #18 AND #23 AND #30 30. Filters from 1999-2021 |

**Supplemental Table 2: Definition of major outcomes**

| Trial/Author | year | MACE definition | Cardiovascular mortality definition | All-cause mortality definition | nonfatal MI definition | repeat revascularization definition |
| --- | --- | --- | --- | --- | --- | --- |
| PRIMA | 2004 | NA | NS | NS | NS | NS |
| HLEP AMI | 2004 | All-cause mortality, recurrent MI, and repeat revascularization | NA | NA | NA | Any revascularization, infarct related artery as well as non-infarct-related period |
| Politi et al. | 2010 | All-cause mortality, recurrent MI, hospitalization for acute coronary syndrome, and revascularization | NS | NS | NA | NS |
| Ghani et al | 2012 | All-cause mortality, recurrent MI, and urgent revascularization | NA | NS | New Q-waves on the ECG or a new CK and CK-MB rise above the ULN. This included peri-procedural infarctions in the invasive treatment arm | NS |
| PRAMI | 2013 | Cardiac death, recurrent MI, and refractory angina | NS | NS | New ECG evidence of ST-segment elevation or left LBBB and angiographic evidence of coronary-artery occlusion | NS |
| Prague 13 | 2015 | Composite of all-cause mortality, recurrent MI, and stroke | NS | NS | NS | NS |
| CROSS-AMI | 2019 | NA | NS | NS | NS | NS |
| COMPLETE | 2019 | CV mortality, MI or ischemia-driven revascularization | All deaths with a clear CVD or unknown cause, will be classified as CVD. However, within CVD deaths, hemorrhagic deaths will be clearly identified | Only deaths due to a documented non-CVD cause (e.g., cancer) will be classified as non-CVD | 3rd Universal definition | Ischemia driven revascularization: Ischemic symptoms consistent with CCS class ≥2 angina despite optimal medical therapy, and 2. PCI or CABG of either the culprit lesion (within 5 mm of the stented segment) associated with the index PCI or a non-culprit lesion that led to enrollment into the trial, and 3. At least one of the following: (i) Positive functional study (ii) New Ischemic ECG changes at rest or with exertion in a distribution consistent with a stenosis; (iii) FFR ≤0.80 |
| CvLPRIT | 2019 | All-cause mortality, recurrent MI, heart failure hospital admission, and repeat revascularization | Cardiovascular death includes any cardiac causes, or other vascular causes | Death from any cause classified as cardiovascular or non-cardiovascular | Hospital admission, or be diagnosed in hospital, with 1 or more of the following: Type 1: (4a, 4b) | 1. Target lesion re-interventions inside the implanted stent or within 5 mm proximally or distally or repeated interventions in the same vessel by percutaneous coronary interventions or by coronary artery bypass graft surgery. 2. PCI to lesions not identified previously. CABG for new symptoms or complications of PCI |
| Compare-Acute | 2020 | All-cause mortality, nonfatal MI, any revascularization and cerebrovascular events | Any death due to immediate cardiac cause (e.g. MI, low-output failure, fatal arrhythmia). Unwitnessed death and death of unknown cause will be classified as cardiac death | All deaths are considered cardiac unless an unequivocal non-cardiac cause can be established. Specifically, any unexpected death even in patients with coexisting potentially fatal non-cardiac disease (e.g. cancer, infection) should be classified as cardiac | Any PCI or bypass surgery for recurrent ischemia that in the investigators opinion can not be delayed for more than 24 hours and is defined by the investigator as a non-elective procedure | All first revascularizations that were elective or urgent and that were clinically indicated or not between the time of the index procedure and follow-up at 12 months |
| DANAMI-3-PRIMULTI | 2015 | All-cause mortality, recurrent MI, and ischemia-driven revascularization | All deaths were judged cardiac-related unless they could be clearly attributed to another cause | All deaths were judged cardiac-related unless they could be clearly attributed to another cause | Typical chest pain was accompanied by a substantial rise in troponins, development of new Q-waves on the electrocardiograph, or both | Ischemia driven revascularization |
| Estevez Loureiro et al. | 2014 | NA | NS | NA | NS | revascularization of any vessel |
| Zhang et al. | 2015 | Recurrent MI, and Cardiac death | NS | NS | NS | NS |
| HAMZA et al. | 2016 | Composite of all-cause mortality, recurrent MI, and ischemia-driven revascularization | NA | NS | NS | ischemia-driven revascularization by PCI or coronary artery bypass grafting (CABG) |
| ZHAO et al. | 2016 | including cardiac death, recurrent myocardial infarction and repeated revascularization of the target‑vessel | NS | NA | NS | NS |
| Tarasov et al. | 2017 | NA | NS | NS | Criteria of re-MI during eighteen hours after index MI was myocardial ischemia with appropriate recurrent ST-segment deviation ≥30 minutes. After eighteen hours re-MI was diagnosed in the case of new Q waves, new left bundle-branch block, and/or appropriate increasing of the level of troponin and/or MB-creatine kinase fraction. | unplanned PCI or coronary artery bypass grafting with the reason for the patients’ implementation such as recurrent symptoms, re-MI or significant ischaemia on stress-testing. |
| Mihnea et al. | 2021 | NA | NA | NS | using the criteria from the fourth universal definition of myocardial infarction and further subdivided them according to the territory involved as it appeared on the ECG | symptom-driven revascularization |

NS: not specified; NA: not applicable; ULN = upper limit of normal; MACE = major adverse cardiac event (s); CVD: cardiovascular death; MI: myocardial infarction; CABG: coronary artery bypass graft; CCS: Canadian Cardiovascular Society; ECG = electrocardiograph; LBBB = left bundle branch block; CV = cardiovascular; PCI = Percutaneous Coronary Intervention; CABG = Coronary Artery Bypass Grafting; FFR = fractional flow reserve; HELP-AMI = HEpacoat for cuLPrit or multivessel stenting for acute myocardial infarction; CROSS-AMI = Complete Revascularization Or streSS Echo in Patients With Multivessel Disease and ST-segment Elevation Acute Myocardial Infarction; Compare-Acute = Comparison Between FFR Guided Revascularization Versus Conventional Strategy in Acute STEMI Patients With MVD; COMPLETE = Complete vs Culprit-only Revascularization to Treat Multi-vessel Disease After Early PCI for STEMI; CvLPRIT = complete versus lesion-only PRimary PCI pilot study; DANAMI-3-PRIMULTI = third danish study of primary PCI in patients with ST-elevation myocardial infarction and multivessel disease: treatment of culprit lesion only or complete revascularization; PRAMI = preventive angioplasty in acute myocardial infarction.

**Supplemental Table 3: Summary of ROB 1.0 Assessment of included Studies**

| Trial/Author | year | Random sequence generation (selection bias) | Allocation concealment (selection bias) | Blinding of participants and researchers (performance bias) | Blinding of outcome assessment (detection bias) | Incomplete outcome data (attrition bias) | Selective reporting (reporting bias) | Other bias |
| --- | --- | --- | --- | --- | --- | --- | --- | --- |
| PRIMA | 2004 | Unclear | Unclear | Low risk | Low risk | Low risk | Low risk | Low risk |
| HLEP AMI | 2004 | Unclear | Unclear | Low risk | Low risk | Low risk | Low risk | Low risk |
| Politi et al. | 2010 | Unclear | Unclear | Unclear | Unclear | Low risk | Low risk | Low risk |
| Ghani et al | 2012 | Low risk | Low risk | Unclear | Low risk | Low risk | Low risk | Low risk |
| PRAMI | 2013 | Low risk | Low risk | Low risk | Low risk | Low risk | Low risk | Low risk |
| Prague 13 | 2015 | Unclear | Unclear | Unclear | Unclear | Unclear | Low risk | Low risk |
| CROSS-AMI | 2019 | Low risk | Low risk | Low risk | Low risk | Low risk | Low risk | Low risk |
| COMPLETE | 2019 | Low risk | Low risk | Low risk | Low risk | Low risk | Low risk | Low risk |
| CvLPRIT | 2019 | Low risk | Low risk | Low risk | Low risk | Low risk | Low risk | Low risk |
| Compare-Acute | 2020 | Low risk | Low risk | Low risk | Low risk | Low risk | Low risk | Low risk |
| DANAMI-3-PRIMULTI | 2015 | Low risk | Low risk | Low risk | Low risk | Low risk | Low risk | Low risk |
| Estevez Loureiro et al. | 2014 | Low risk | Unclear | Unclear | Unclear | Low risk | Low risk | Low risk |
| Zhang et al. | 2015 | Low risk | Unclear | Unclear | Unclear | Low risk | Low risk | Low risk |
| HAMZA et al. | 2016 | Unclear | Unclear | Unclear | Unclear | Low risk | Low risk | Low risk |
| ZHAO et al. | 2016 | Low risk | Unclear | Unclear | Unclear | Low risk | Low risk | Low risk |
| Tarasov et al. | 2017 | Unclear | Unclear | Low risk | Low risk | Low risk | Low risk | Low risk |
| Mihnea et al. | 2021 | Unclear | Unclear | Unclear | Unclear | Low risk | Low risk | Low risk |

ROB = Cochrane risk of bias tool; HELP-AMI = HEpacoat for cuLPrit or multivessel stenting for acute myocardial infarction; CROSS-AMI = Complete Revascularization Or streSS Echo in Patients With Multivessel Disease and ST-segment Elevation Acute Myocardial Infarction; Compare-Acute = Comparison Between FFR Guided Revascularization Versus Conventional Strategy in Acute STEMI Patients With MVD; COMPLETE = Complete vs Culprit-only Revascularization to Treat Multi-vessel Disease After Early PCI for STEMI; CvLPRIT = complete versus lesion-only PRimary PCI pilot study; DANAMI-3-PRIMULTI = third danish study of primary PCI in patients with ST-elevation myocardial infarction and multivessel disease: treatment of culprit lesion only or complete revascularization; PRAMI = preventive angioplasty in acute myocardial infarction.

**Supplemental Figure 1: Quality assessment of each study**


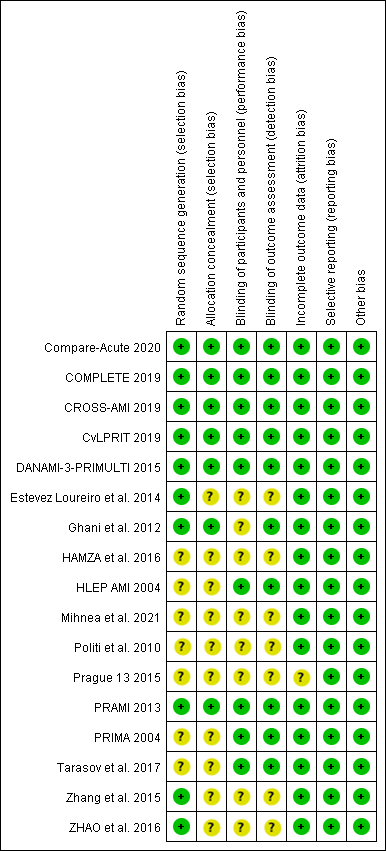


**Supplemental Figure 2: Meta-regression of follow-up months in two groups of revascularization**


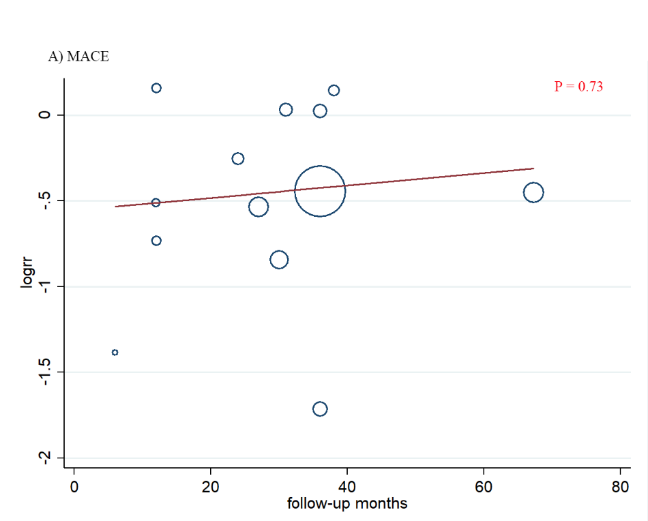

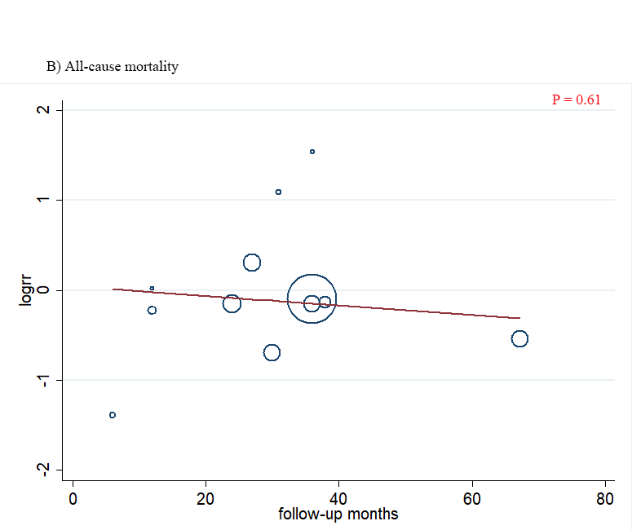


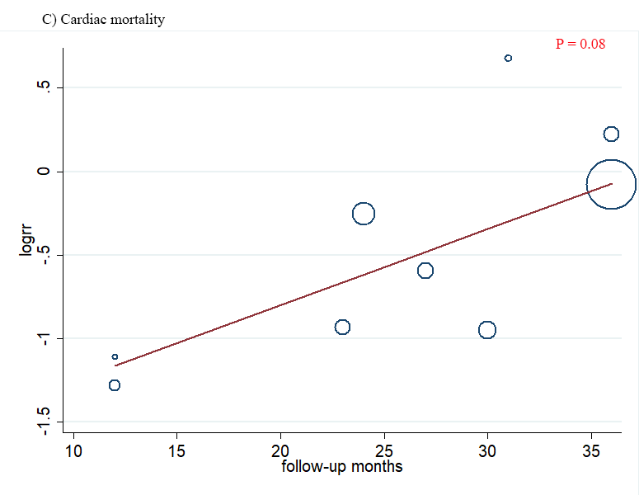

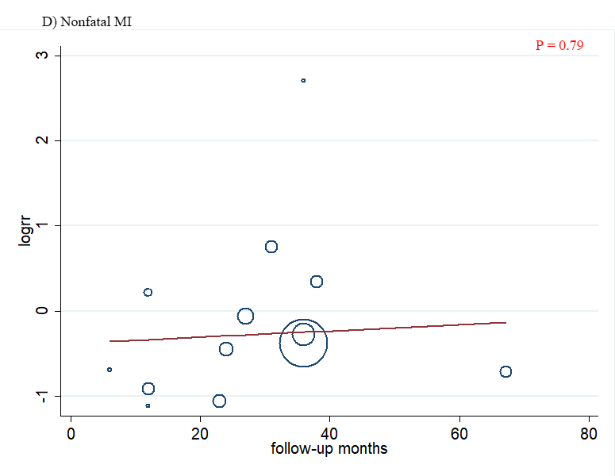


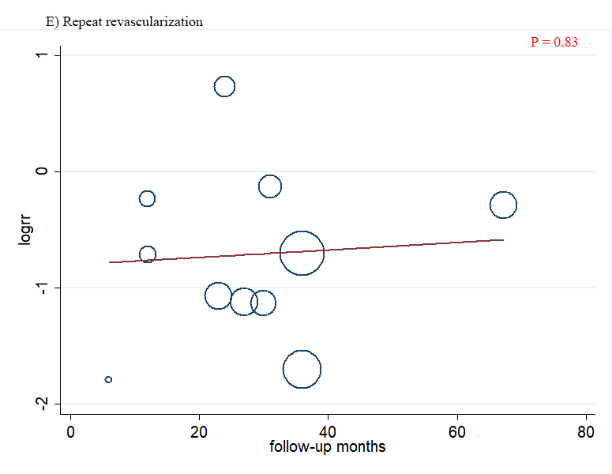

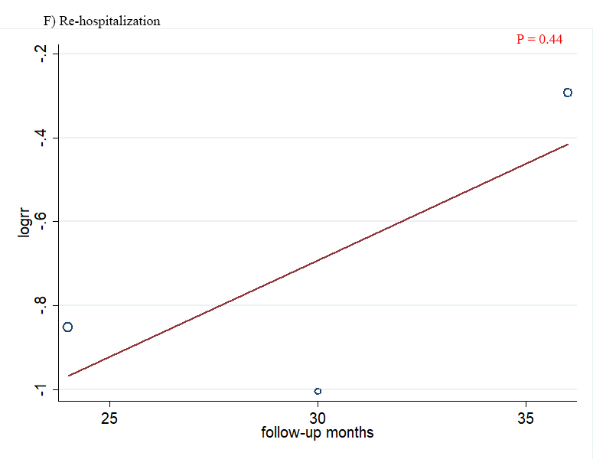


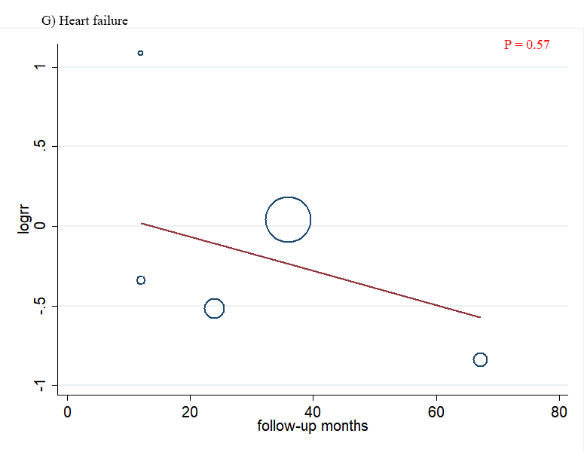

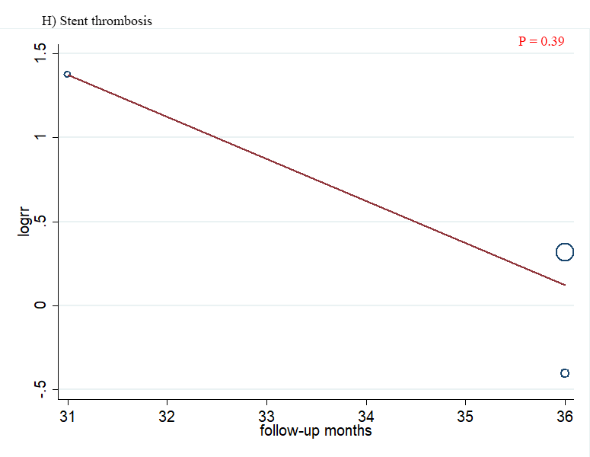


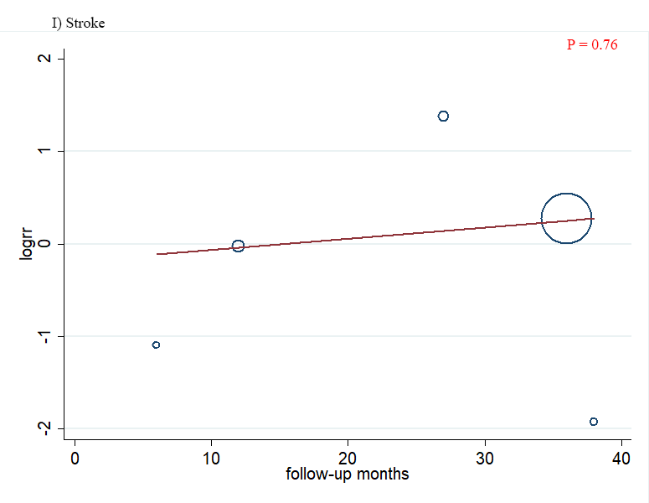

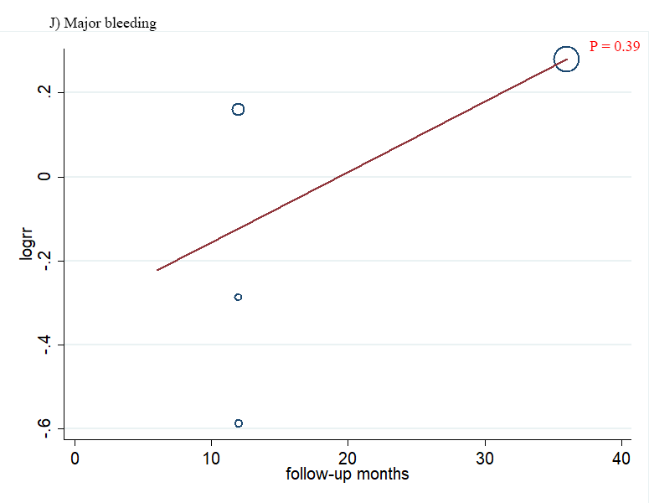


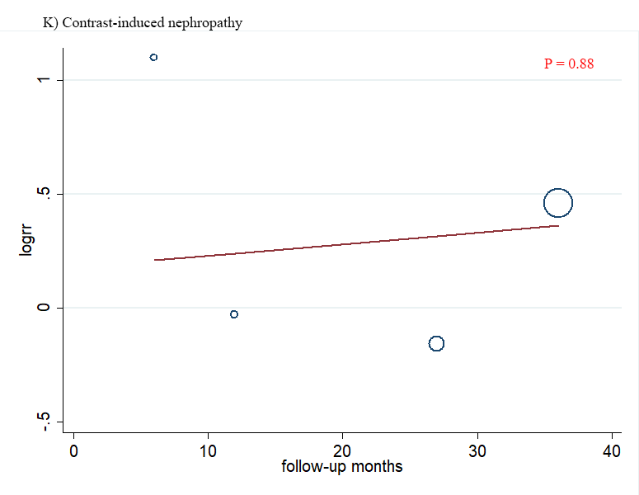


**Supplemental Table 4: Summary of publication bias for all outcomes**

| outcome | OR | 95 CI% | P value |
| --- | --- | --- | --- |
| MACE | 0.04 | -2.11-2.19 | 0.97 |
| All-cause mortality | 0.01 | -0.94-0.96 | 0.98 |
| Cardiac mortality | -0.82 | -2.12-0.47 | 0.18 |
| Nonfatal MI | 0.48 | -0.86-1.82 | 0.45 |
| Repeat revascularization | 1.84 | -2.32-5.99 | 0.34 |
| Re-hospitalization | -6.26 | -104.35-91.84 | 0.57 |
| Heart failure | -0.54 | -3.02-1.94 | 0.54 |
| Stent thrombosis | 0.65 | -33.62-34.92 | 0.85 |
| Stroke | -0.57 | -2.75-1.61 | 0.47 |
| Major bleeding | -1.39 | -3.56-0.78 | 0.11 |
| Contrast-induced nephropathy | -0.18 | -3.97-3.61 | 0.85 |

OR = odds ratio; CI = confidence interval; MACE = major adverse cardiac event (s); MI = myocardial infarction;

**Supplemental Figure 3: Pairwise meta-analysis summary of clinical outcomes**

**
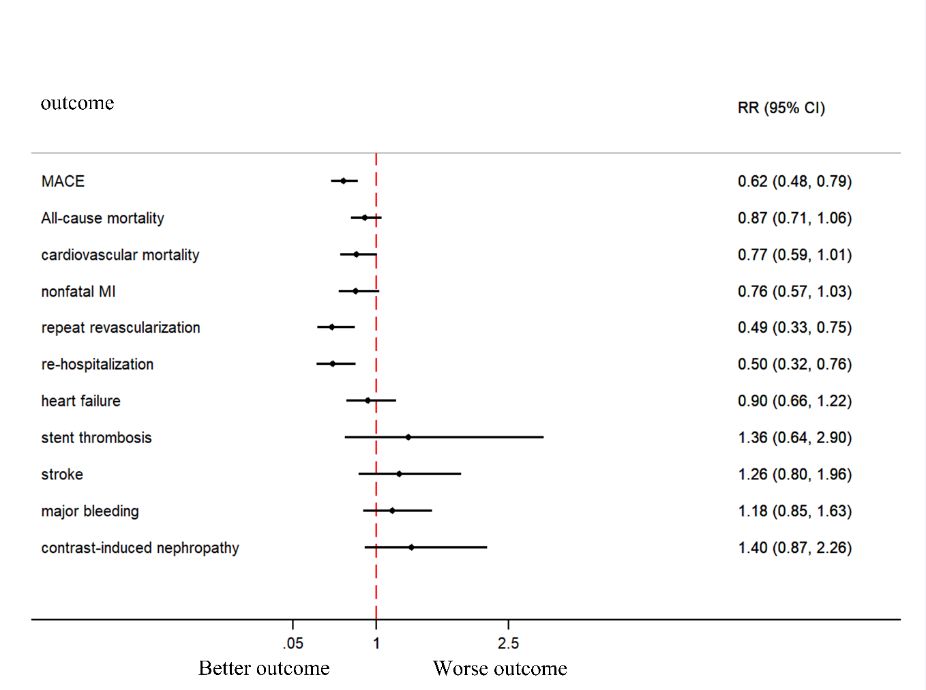
**

Summary plot of RR and the corresponding 95% CI for outcomes of pairwise meta-analysis. MACE = major adverse cardiac event(s); CI = confidence interval; RR = risk ratio; MI = myocardial infarction.

**Supplemental Figure 4: Sensitivity analysis**

1. **Excluding each study successively**

**B) Excluding trials with relatively small sample size (i.e., trials with included patients ≤100 in each group)**


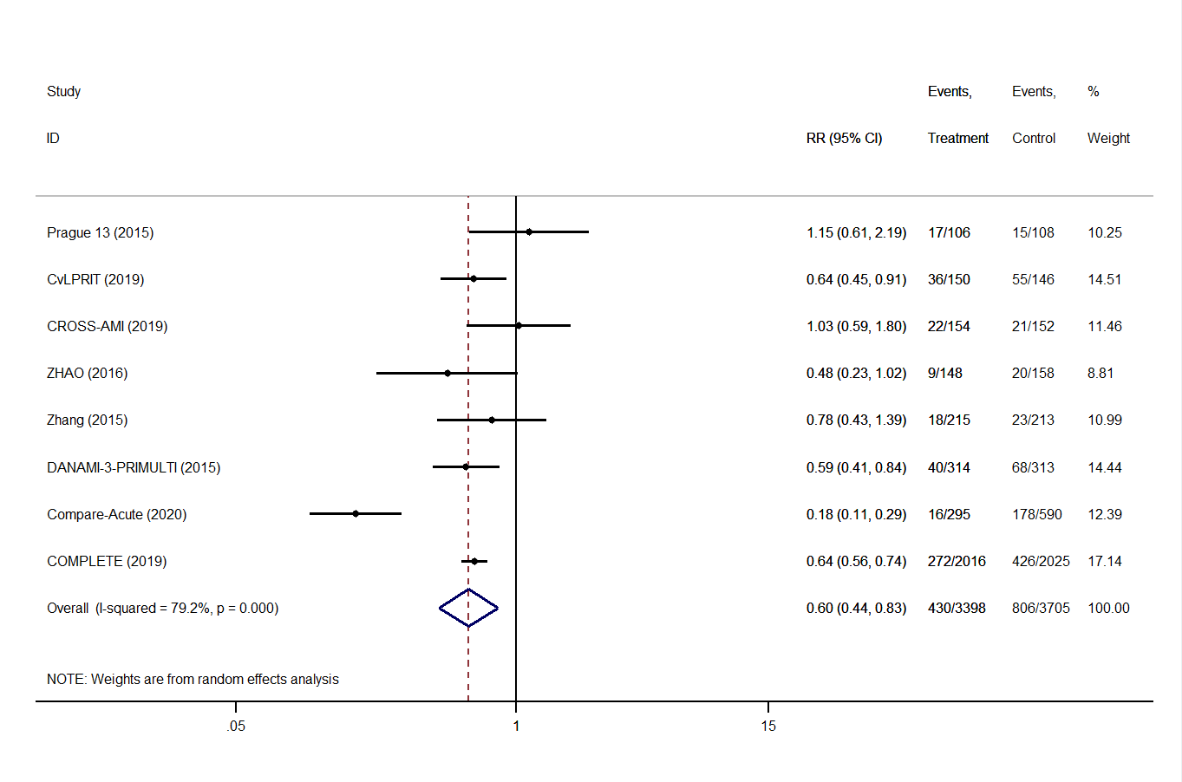


**C) Excluding older trials (i.e., trials before 2014)**


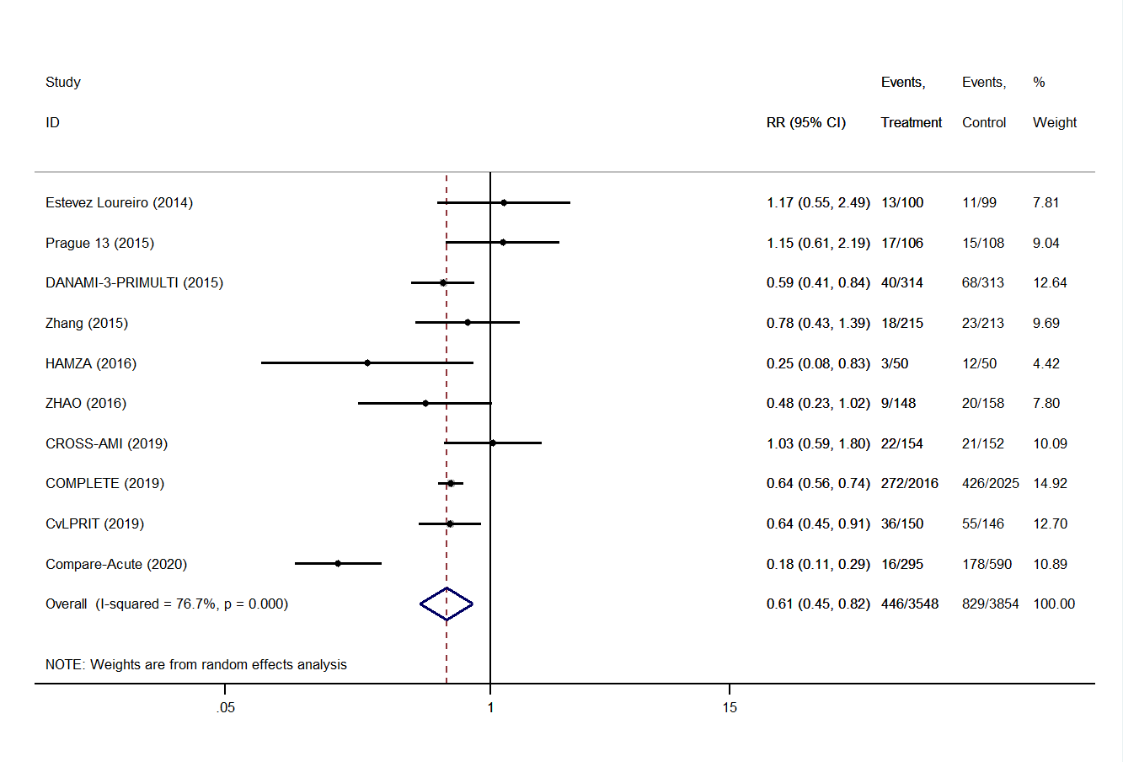


**Supplemental Figure 5: Trace and density plots of all outcomes**

1. **MACE**


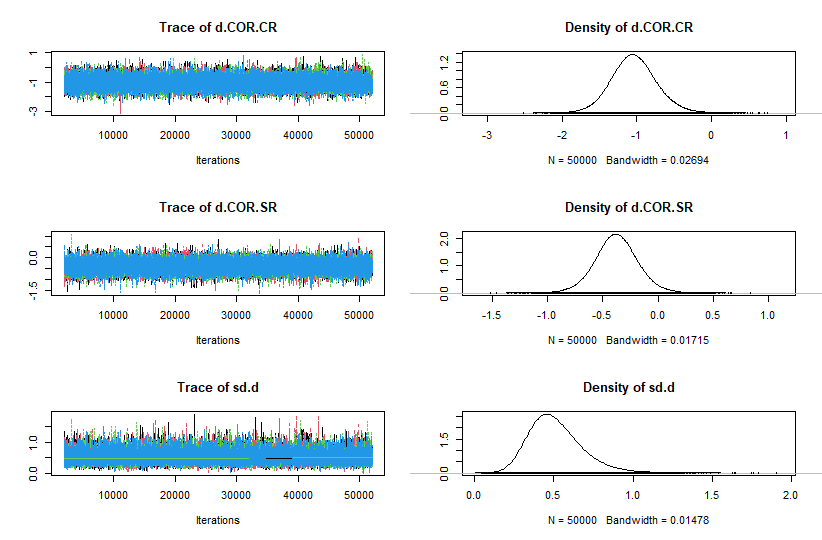


1. **All-cause mortality**


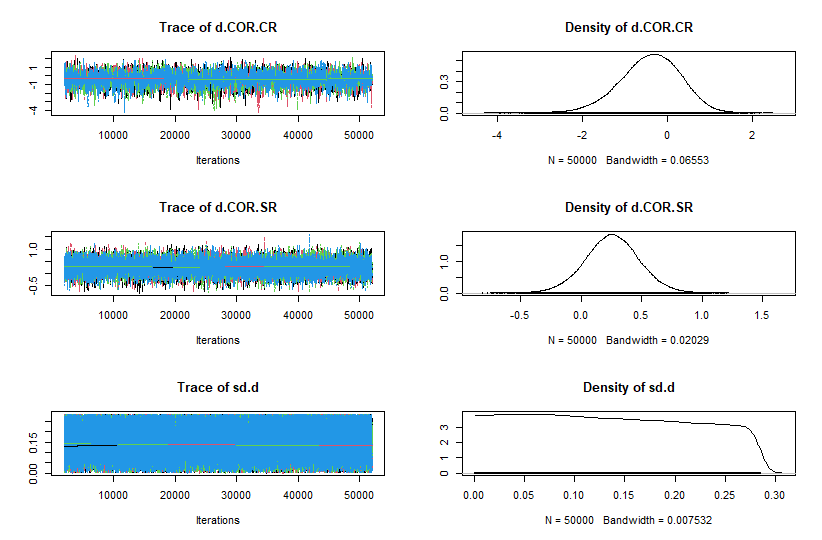


1. **Cardiac mortality**


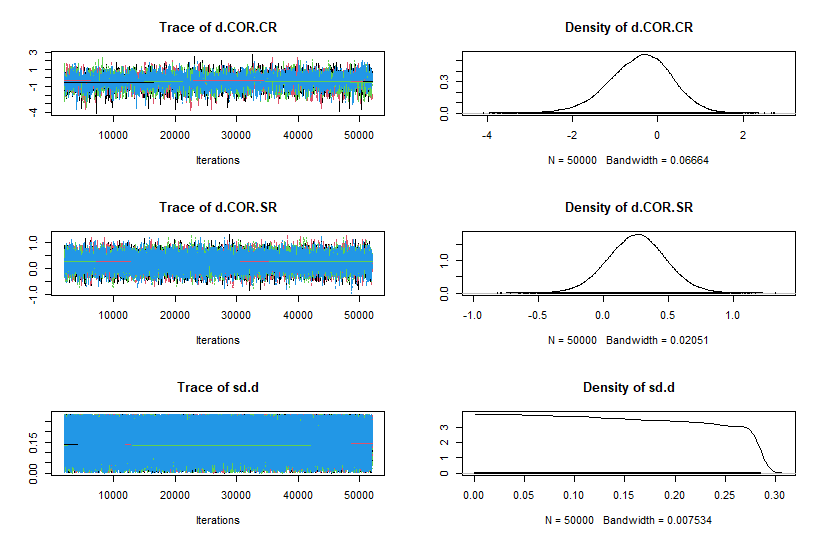


1. **Nonfatal MI**


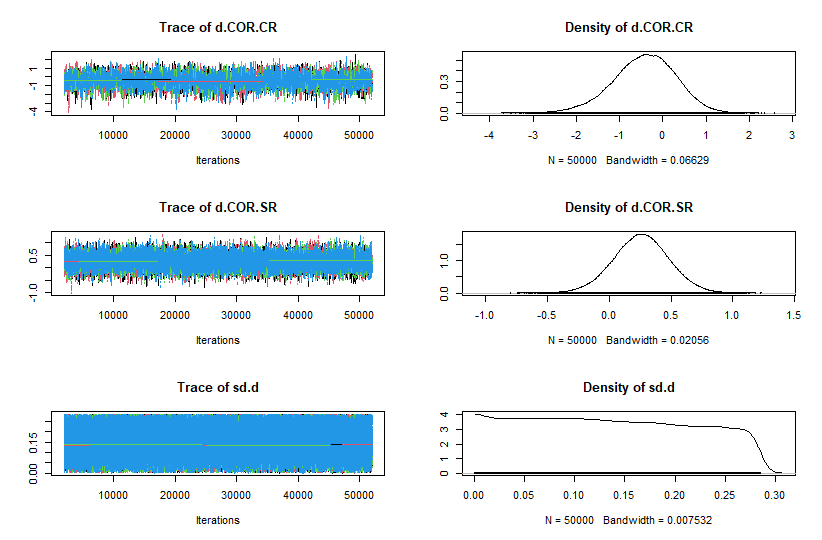


1. **Repeat revascularization**


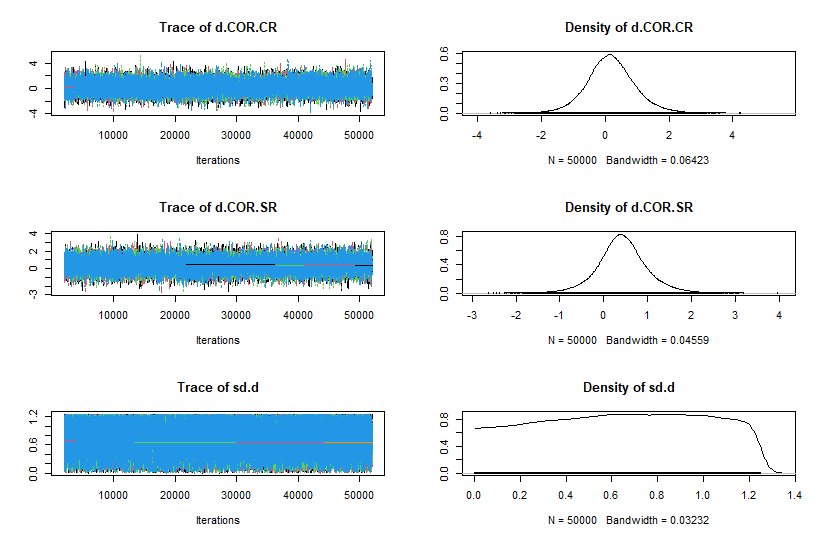


1. **Major bleeding**


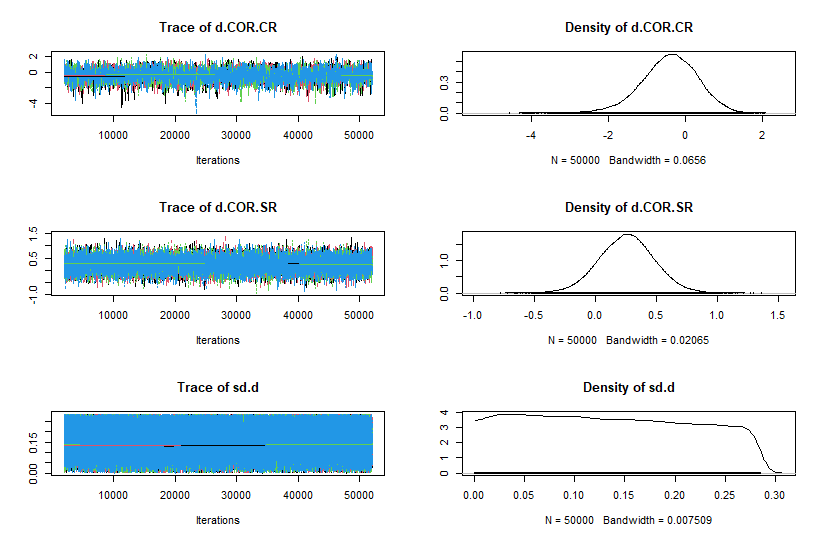


1. **Stent thrombosis**


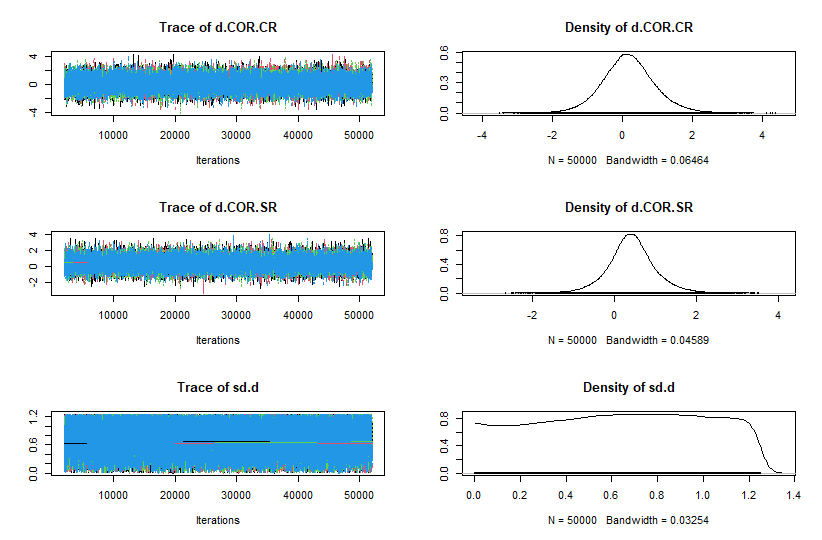


**Supplemental Figure 6: Local inconsistency analysis of all outcomes**

**A) MACE**


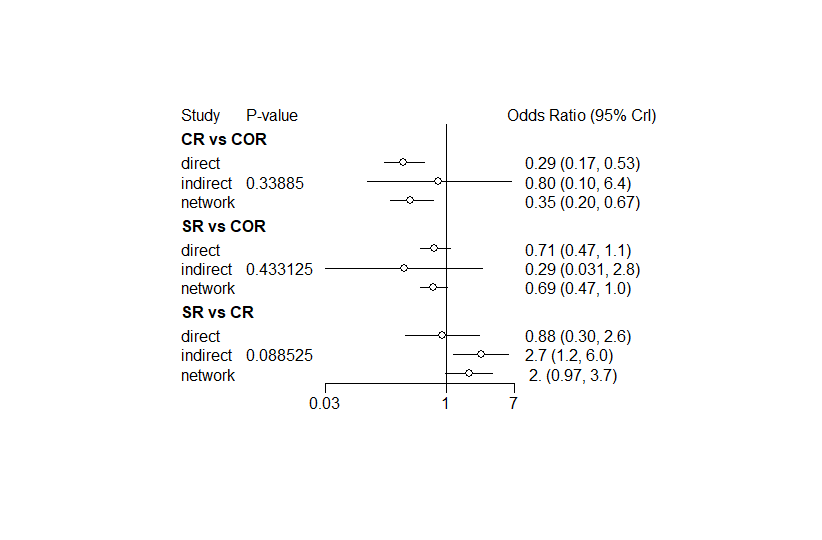


**B) All-cause mortality**


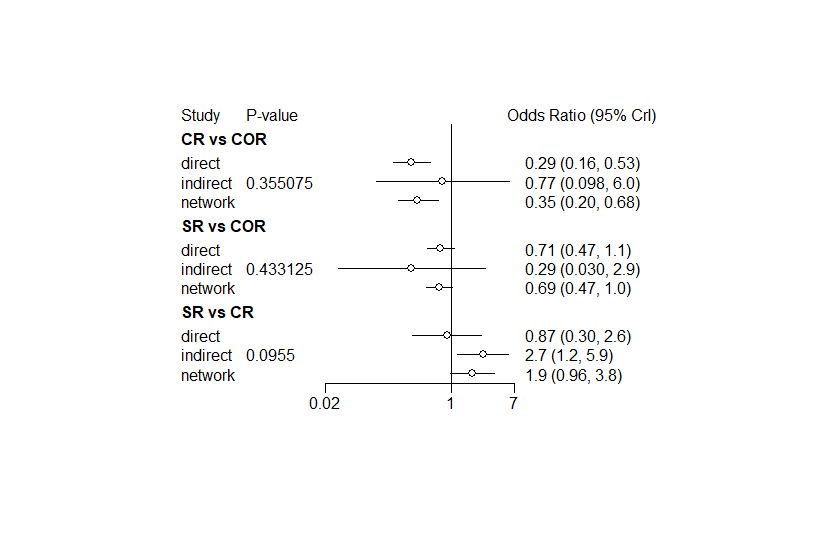


**C) Cardiac mortality**


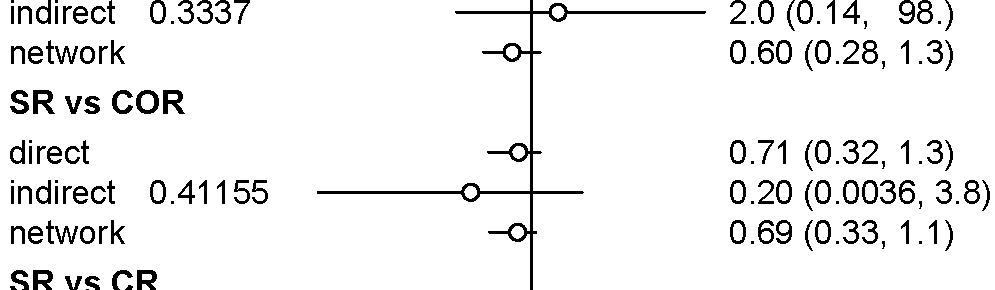


**D) Nonfatal MI**


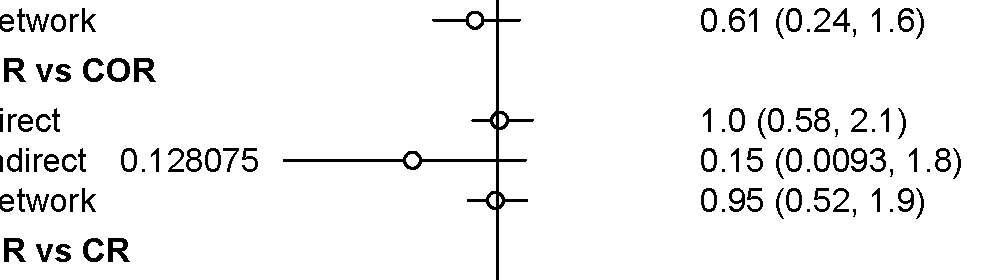


**E) repeat revascularization**


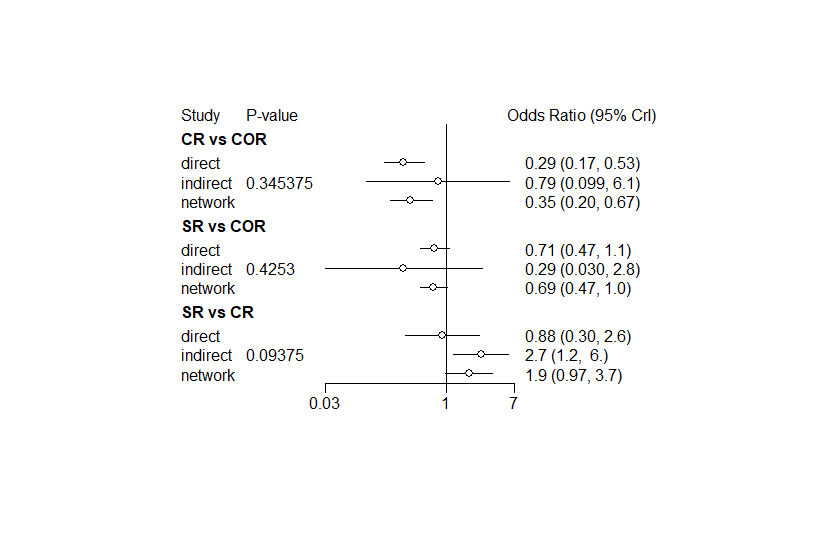


**F) Major bleeding**


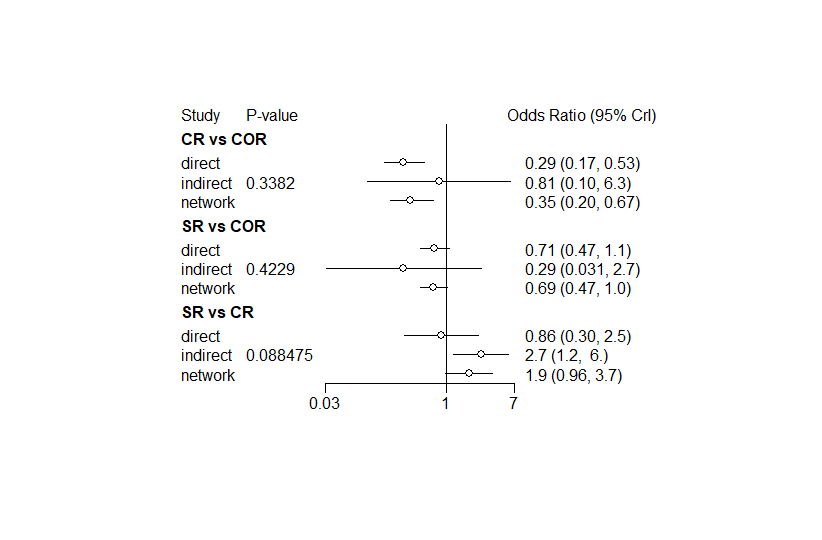


**G) Stent thrombosis**


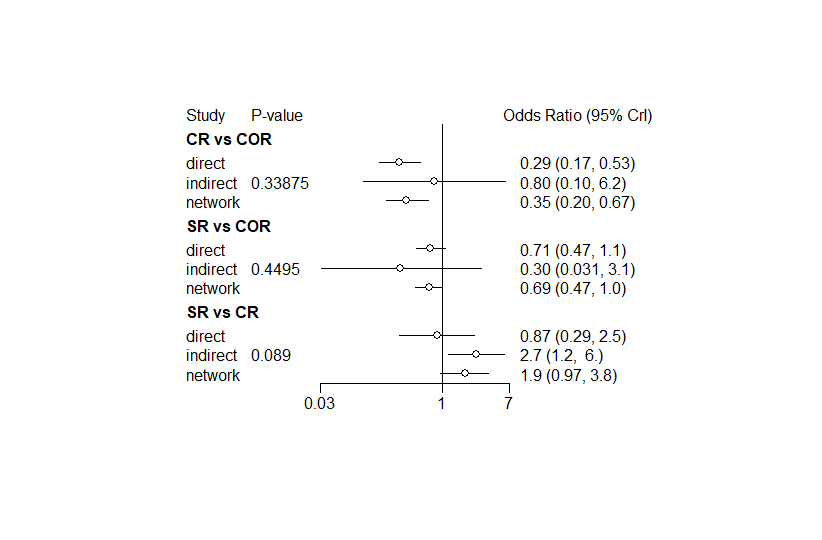

Supplement: Supplementary file 1 [file Data_Sheet_1.docx]
